# Selective Enrichment of Fluorescent Nanodiamonds by Stimulated Recoil Forces

**DOI:** 10.1021/acsnano.5c22759

**Published:** 2026-03-26

**Authors:** Yoshiki Saito, Takao Horai, Yoshiki Umekawa, Ryosuke Shimono, Yoshihiro Tomoi, Takuya Matsuda, Yuto Makino, Yosuke Minowa, Hajime Ishihara, Masaaki Ashida

**Affiliations:** † Graduate School of Engineering Science, 154366 The University of Osaka, 1-3 Machikaneyama-cho, Toyonaka, Osaka 560-8531, Japan; ‡ 57903 Daicel Corporation, 1239 Shinzaike, Aboshi-ku, Himeji, Hyogo 671-1283, Japan; § Department of Physics, 12918 Kyoto University, Kitashirakawa-Oiwake-cho, Sakyo-ku, Kyoto 606-8501, Japan; ∥ Research Organization of Science and Technology, Ritsumeikan University, 1-1-1, Nojihigashi, Kusatsu, Shiga 525-8577, Japan; ⊥ SANKEN, The University of Osaka, 8-1 Mihogaoka, Ibaraki, Osaka 567-0047, Japan; # Ritsumeikan Semiconductor Application Research Center (RISA), Ritsumeikan University, 1-1-1 Nojihigashi, Kusatsu, Shiga 525-8577, Japan

**Keywords:** optical force, optical sorting, nanodiamonds, stimulated recoil force, detonation nanodiamonds, SiV-centers in nanodiamonds

## Abstract

Detonation nanodiamonds containing silicon-vacancy (SiV) centers (SiV-DNDs) exhibit spectrally sharp optical transitions and are promising nanoscale emitters. After purification and oxidative postprocessing, SiV-DNDs are obtained with a mean particle diameter of ∼10 nm, a size scale at which single-color-center occupancy per particle may be expected. Yet, practical applications require selective enrichment from mixtures that also contain undoped nanodiamonds. Conventional separation methods lack sufficient selectivity, and resonant absorption-based optical sorting is fundamentally constrained by excited-state saturation, rendering it ineffective in the single-color-center regime. Building on our recent theoretical predictions that stimulated emission can generate a dissipative optical force beyond this limit, we demonstrate that the stimulated recoil force (SRF) provides a scalable mechanism for emission-line-selective manipulation of nanodiamonds in liquid. Using a glass capillary with counter-propagating pump beams and a manipulation beam resonant with SiV emission, we observe millimeter-scale downstream depletion and upstream enrichment of SiV-DNDs, while a spectrally distinct, off-resonant fluorescent nanodiamond population remains unchanged. The magnitude and spatial extent of the transport show that SRF overcomes Brownian diffusion and enables long-range, species-selective transport under realistic conditions. To identify the physical mechanism, we perform complementary glass-cell experiments under a well-defined focusing geometry and compare the observed enrichment with optical-force calculations based on density-matrix dynamics and Brownian-dynamics simulations. Qualitative agreement supports SRF as the dominant dissipative contribution responsible for the transport. These results demonstrate practical, emission-energy-selective optical sorting of fluorescent nanodiamonds and define design principles for extending this approach to capillaries, microfluidic systems, and other fluorescent nanomaterials.

## Graphical abstract

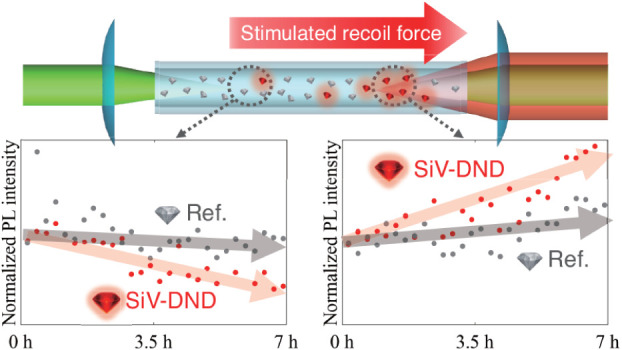

Fluorescent nanodiamonds (F-NDs) incorporating group-IV vacancy centers are promising nanoscale emitters for quantum sensing, bioimaging, and integrated photonics because of their bright, spectrally sharp zero-phonon lines (ZPLs) and excellent photostability.^1–32^ Recently, silicon-vacancy (SiV) and germanium-vacancy (GeV) center-containing detonation nanodiamonds (DNDs) have been successfully synthesized by a detonation process; hereafter, we refer to these samples as SiV-DNDs and GeV-DNDs, respectively.^17,33^ By combining detonation synthesis with subsequent purification and oxidative postprocessing, this route yields SiV-DNDs and GeV-DNDs with mean particle diameters of around 10 nm.^34,35^ Bright and spectrally sharp room-temperature ZPL emission has been reported for such samples, with the postprocessing steps further improving the optical properties and sample quality.^34–36^ The detonation process enables the production of NDs in large quantities,^37,38^ opening a pathway toward practical applications of SiV-NDs and GeV-NDs. However, detonation synthesis yields mixtures in which undoped DNDs are coproduced with fluorescent DNDs.^17,36^ Moreover, given the ∼10 nm particle size, each ND is ideally expected to contain single color center (see Materials and Methods and Supporting Information, Sec. S5).^36,39^ Highly selective enrichment of F-NDs from the much larger background of undoped particles is therefore essential for scalable deployment of these quantum-grade nanomaterials.

Optical-force manipulation provides a promising route toward such selective enrichment. The optical force comprises a gradient force, which confines particles via spatial intensity variations, and a dissipative force arising from photon momentum transfer, which enables directed transport.^40–55^ Dissipative-force schemes exploiting resonant absorption have been investigated and demonstrated,^56–62^ including selective transport of nanodiamonds with or without nitrogen-vacancy (NV) centers near tapered fibers and in narrow channels.^59,61^ In these NV-ND-based experiments, the NDs typically hosted hundreds to thousands of NV centers per particle, so that the total absorption cross-section, and hence the absorption force, was large enough to enable selective transport. By contrast, the SiV-DNDs and GeV-DNDs considered here ideally contain only on the order of single group-IV center per DNDs, so that the absorption force is intrinsically reduced by orders of magnitude. For such ∼10 nm single-emitter DNDs, the excited-state population also saturates at relatively low optical intensities, which further limits the usable absorption-based force and prevents efficient manipulation by resonant absorption alone.^63^

The stimulated recoil force (SRF) offers a fundamentally different dissipative mechanism.^64^ SRF originates from the momentum of photons emitted via stimulated emission. Stimulated emission rapidly depletes the excited state and repopulates the ground state, thereby avoiding saturation and allowing SRF to produce strong optical forces even under intense optical driving. Furthermore, SRF derives its selectivity from the emission transition itself, enabling discrimination of nanoscale emitters based on their emission-line energies rather than their absorption spectra. Our recent theoretical work predicted that SRF can surpass resonant absorption forces and enable emission-line-selective transport of single-emitter NDs under realistic liquid-phase conditions.^65^ While these predictions identified a concrete route beyond the long-standing saturation limit of optical manipulation, their experimental realization has remained highly challenging, yet promised to establish a new stage toward quantum-mechanical, emission-based sorting of nanomaterials if achieved.

In this work, we demonstrate SRF-assisted, long-range, species-selective transport of SiV-DNDs in liquid. In commercial glass capillaries with counter-propagating pump beams and a manipulation beam resonant with the SiV-ZPL, we observe millimeter-scale depletion of SiV-DNDs downstream and enrichment near the input, while GeV-DNDs remain essentially unchanged. This behavior agrees with the theoretical trends predicted for SRF-driven transport of SiV- and GeV-DNDs.^65^ To unambiguously identify the underlying mechanism, we further conduct glass-cell experiments under a well-defined focusing geometry and directly compare the observed enrichment with optical-force calculations and Brownian-dynamics simulations. The qualitative agreement confirms that SRF is the dominant dissipative force responsible for the emission-wavelength-selective transport.

Together, these results establish SRF as a practical and scalable mechanism for selectively manipulating quantum-grade NDs in liquid environments. They demonstrate that stimulated-emission-based optical forces can displace single-emitter NDs over millimeter distances and provide quantitative design principles for emission-energy-selective optical sorting in capillaries, microfluidic channels, and other fluorescent nanomaterial systems.

## Results and Discussion

### Selective Enrichment in a Capillary Configuration

To demonstrate that emission-selective optical manipulation can be extended to a practical, millimeter-scale geometry, we performed experiments in a liquid-filled glass capillary [Figure 1a]. The capillary was irradiated by two counter-propagating pump beams to excite both SiV and GeV centers while canceling the absorption force along the capillary axis, while providing the excited-state population required for the SRF cycle. A third beam (manipulation beam) was injected from one end and tuned either on resonance with the SiV-ZPL (to enable stimulated-emission cycles and generate SRF on SiV-DNDs) or detuned/off (controls).

**Figure 1:**
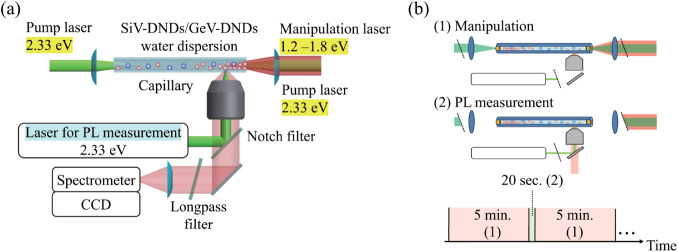
(a) Schematic of the capillary configuration with counter-propagating pump beams and a manipulation beam. (b) Timing sequence for the capillary PL measurements, showing alternating irradiation and readout periods.

Because GeV centers are far off-resonant from the SiV-resonant manipulation condition, GeV-DNDs serve as an in situ off-resonant reference population during the capillary transport measurements. Photoluminescence (PL) spectra were recorded at two axial positions along the capillary (positions #1 and #2), allowing us to monitor enrichment/depletion over millimeter distances. Further details of capillary preparation, optical parameters, beam delivery, and the irradiation/readout sequence are provided in Materials and Methods.

#### Spectral Characteristics and Concentration Calibration in the Capillary

Figure 2a shows a PL spectrum of the mixed SiV-DND/GeV-DND water dispersion recorded in the capillary configuration, featuring ZPLs at 1.68 eV (SiV) and 2.06 eV (GeV) together with broadband emission and Raman bands from water. The broadband background (gray) is attributed to NV^0/–^ centers and surface defects.^36^ For quantitative analysis, this background was approximated by a third-order polynomial and subtracted, isolating the SiV-ZPL, GeV-ZPL, and water Raman components. We then varied the mass concentration and evaluated SiV-DND/H_2_O and GeV-DND/H_2_O, defined as the integrated ZPL intensities normalized by the water Raman intensity. As shown in Figure 2b, both quantities scale almost linearly with concentration, confirming that they can be used as proxies for the SiV-DND and GeV-DND concentrations in the capillary measurements.

**Figure 2:**
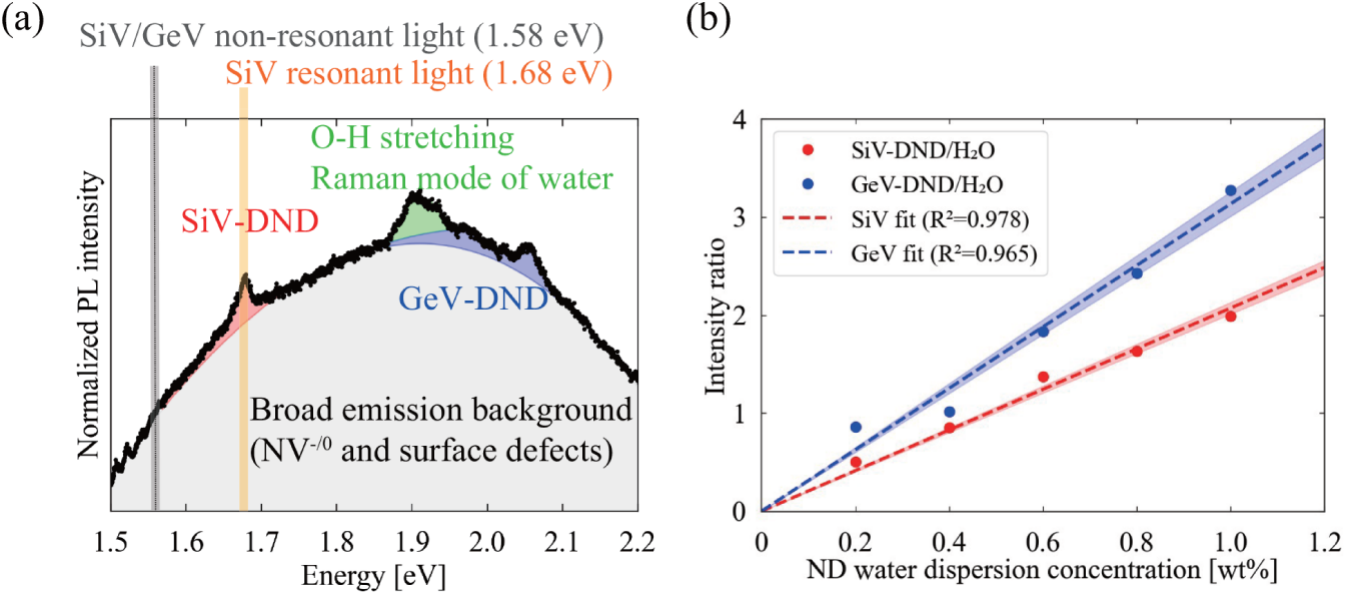
(a) PL spectrum of the mixed SiV-DND/GeV-DND water dispersion recorded in the capillary configuration, showing ZPLs from SiV and GeV centers and Raman scattering from water. The broadband background attributed to NV centers and surface defects is indicated in gray.^36^ (b) Normalized intensities SiV-DND/H_2_O and GeV-DND/H_2_O as a function of concentration in the capillary geometry. The shaded areas indicate the ± 1σ range.

#### Capillary-Scale Redistribution of SiV-DNDs

To quantify the capillary-scale selectivity in a form consistent with the definition used throughout this work, we introduce the enrichment factor

1 $$E\left(t\right)=\frac{\left(I_{\mathrm{SiV}}/I_{\mathrm{GeV}}\right)\left(t\right)}{\left(I_{\mathrm{SiV}}/I_{\mathrm{GeV}}\right)\left(t=0\right)}$$

where *I*_SiV_ and *I*_GeV_ denote the background-subtracted, Raman-normalized ZPL intensities (SiV-DND/H_2_O and GeV-DND/H_2_O, respectively).

Figure 3a shows the time evolution of the enrichment factor in the capillary under counter-propagating pump irradiation and a SiV-resonant manipulation beam. At position #1 (1 mm from the input end), the enrichment factor increases to approximately 1.4 after 7 h, whereas at position #2 (3 mm), it decreases to approximately 0.6. The inset of Figure 3a presents the corresponding raw traces of SiV-DND/H_2_O and GeV-DND/H_2_O used to evaluate the enrichment factor. At position #1, the SiV-DND/H_2_O signal increases to about 150% of its initial value after 7 h, while at position #2, it decreases to roughly 50%. In contrast, the GeV-DND/H_2_O signal remains essentially constant at both positions. These results indicate enrichment of SiV-DNDs near the input and depletion several millimeters downstream, whereas the GeV-DNDs show no redistribution. The typical measurement uncertainty of the PL ratios in this geometry is ∼5% (Figure S6). For comparison, absorption-force-based enrichment of the same SiV-DND samples in a similar capillary geometry was reported to reach only ∼1.2 even after 40 h of irradiation.^63^ In that absorption-force experiment, only a high-power manipulation beam (∼3 W) was applied, so that the excitation was already in the saturated regime and the absorption force was effectively close to its maximum attainable value. The clear contrast in enrichment efficiency therefore supports the view that, in the single-emitter regime, absorption-force-based transport is fundamentally constrained by excited-state saturation, whereas SRF is less susceptible to saturation because stimulated emission rapidly depletes the excited state and maintains the SRF cycle under strong driving. Moreover, while the absorption-force scheme pushes particles along the manipulation-beam propagation direction, the present experiment exhibits transport in the opposite direction (toward the manipulation-beam input), which is most naturally explained by an SRF-dominated mechanism.

**Figure 3:**
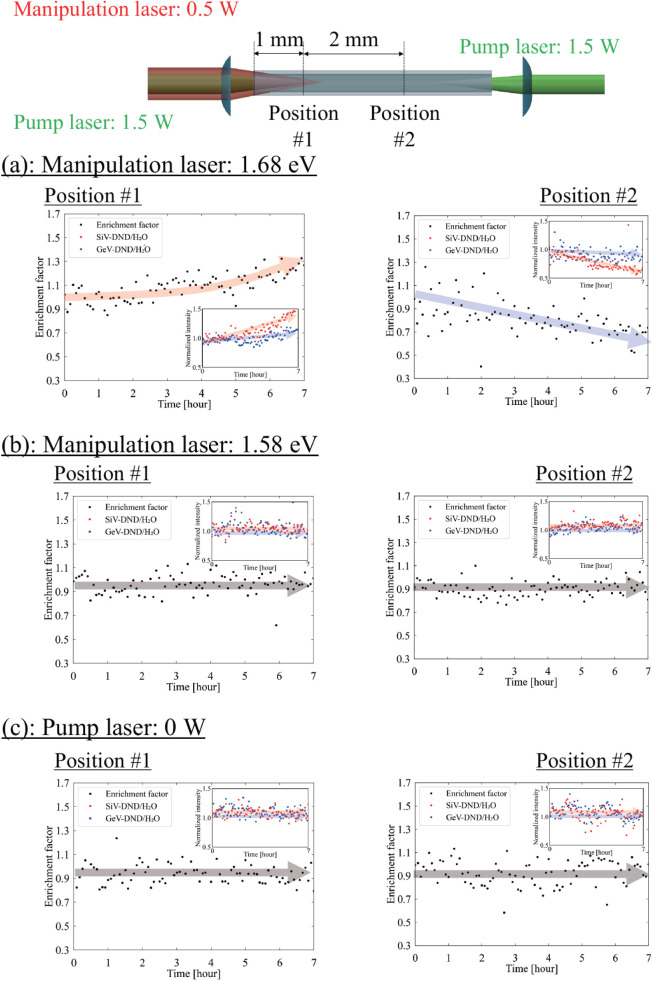
Time evolution of the enrichment factor in the capillary, evaluated at two observation positions. The enrichment factor is defined as *E*(*t*) = [(*I*_SiV_/*I*_GeV_)­(*t*)]/[(*I*_SiV_/*I*_GeV_)­(*t* = 0)], where *I*_SiV_ and *I*_GeV_ denote SiV-DND/H_2_O and GeV-DND/H_2_O, respectively. (a) On-resonance condition: pump on (2.33 eV, 3.0 W in total, counter-propagating) and SiV-resonant manipulation (1.68 eV, 500 mW) produce an increase at position #1 (1 mm from the manipulation-beam input end) and a decrease at position #2 (3 mm). (b) Detuned control (manipulation at 1.58 eV) and (c) pump-off control (pump power 0 W) show no change in *E*(*t*). Insets in each panel show the corresponding time traces of SiV-DND/H_2_O and GeV-DND/H_2_O used to compute *E*(*t*). The solid arrows are guides to the eye.

To determine whether heating, adsorption, or pump-induced convection could produce similar behavior, we performed two control measurements in which SRF is strongly suppressed: (i) manipulation-only irradiation and (ii) pump-on with an off-resonant manipulation beam. In the off-resonant case, the manipulation photon energy was set to 1.58 eV, corresponding to a detuning of about 0.10 eV (∼100 meV) below the SiV^–^-ZPL, i.e., well beyond the inhomogeneous ZPL line width of order ∼30 meV and the associated phonon sideband that extends over ∼60 meV. This large detuning strongly suppresses the spectral overlap between the manipulation beam and the SiV emission, and hence the SRF contribution. Under both control conditions, the SiV-DND and GeV-DND signals at positions  #1 and #2 remained constant within the experimental uncertainty, i.e., *E*(*t*) stayed close to 1, and no depletion or enrichment was observed (Figure 3b,c). These controls show that nonresonant mechanisms alone cannot account for the selective redistribution.

We further examined whether absorption-induced heating or resulting convection could account for the observed millimeter-scale redistribution. As detailed in the Supporting Information (Figure S7), the SiV-ZPL shift was used as an optical thermometer.^16^ The maximum inferred temperature rise at either observation point was Δ*T* ≲ 0.7 K, indicating that substantial thermal gradients or buoyancy-driven flows across the ∼2  mm separation between positions #1 and #2 are unlikely. While minor residual flows cannot be completely ruled out, the SI analysis shows that thermal effects are too small to account for the observed, selective transport, further supporting an SRF mechanism.

To quantify the reproducibility of the capillary measurements, we repeated the experiment on several independently prepared capillaries and evaluated the final enrichment factor at *t* = 7 h, *E*(*t* = 7 h), using eq 1. Based on repeated PL measurements of a spatially homogeneous mixed dispersion, the experimental uncertainty of the ratio was determined to be ± 5% (1σ, Figure S6). We therefore defined a run as “successful” when *E*(*t* = 7 h) exceeded this ± 1σ uncertainty band, i.e., when *E*(*t* = 7 h)>1.05. In total, we performed eight runs under nominally identical conditions, yielding *E*(*t* = 7 h) in the range 1.00–2.02 (see Figure S9 for the full distribution). Under the above criterion, five out of eight runs (63%) were categorized as successful, exhibiting *E*(*t* = 7 h) = 1.19–2.02, while the remaining runs showed no statistically significant redistribution within the ± 5% band. We attribute this spread primarily to the sensitivity of the multimode capillary geometry to sealing conditions, alignment, and variations in the internal optical mode structure.

The average result of the present experiment is in very good agreement with the theoretical predictions in ref. 65, which demonstrates that SRF-assisted, long-range, species-selective transport is indeed feasible and effective. However, commercial capillaries inevitably exhibit variations in geometry and multimode propagation, leading to ill-defined internal optical-field distributions. Because such uncontrolled features make it neither meaningful nor reliable to assume a specific optical mode for each individual capillary and perform simulations tailored to that assumed mode, we instead carried out glass-cell experiments under a well-defined focusing geometry. This controlled configuration allows quantitative and model-independent comparison with optical-force calculations and Brownian-dynamics simulations, thereby providing a much clearer understanding of the physical mechanism underlying SRF-assisted transport.

### Selective Enrichment in a Glass-Cell Configuration

#### Glass-Cell Configuration

To identify the physical mechanism responsible for the selective transport in a well-defined optical-field geometry, we performed complementary experiments in a closed glass cell [Figure 4a]. In the standard configuration, two counter-propagating pump beams excited the color centers while largely canceling the absorption force, and a focused manipulation beam tuned to the SiV-ZPL was applied from the top to induce SRF on SiV-DNDs.

**Figure 4:**
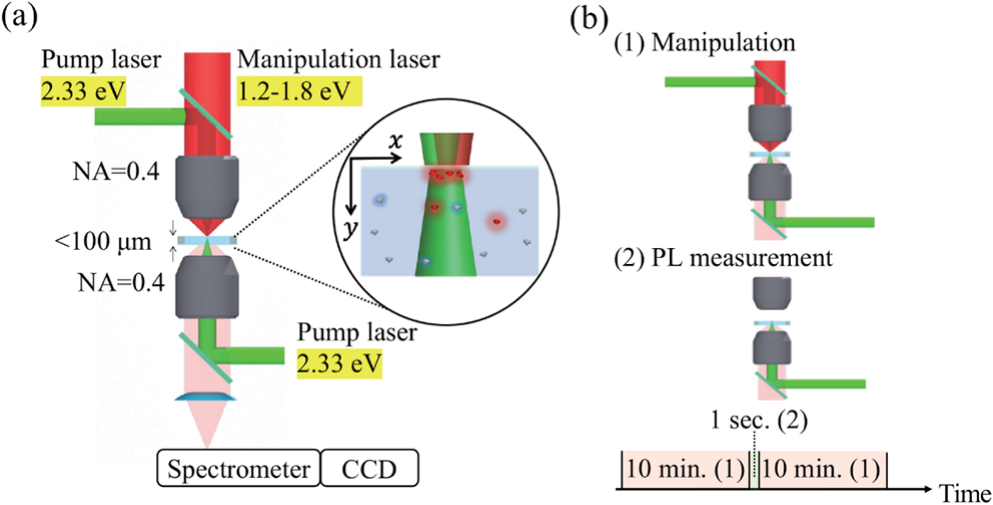
(a) Schematic of the glass-cell configuration with counter-propagating pump beams and a focused manipulation beam. (b) Timing sequence for the glass-cell PL measurements.

Selective enrichment was evaluated by PL measurements. Control experiments (pump-only and detuned manipulation) were conducted under comparable optical loading to separate resonance-enabled SRF contributions from nonresonant effects. Experimental details of cell preparation, focusing geometry, beam parameters, and the measurement sequence are provided in the Materials and Methods.

#### Spectral Characteristics and Concentration Calibration in the Glass Cell

Additional spectral characterization and the concentration calibration specific to the glass-cell experiment are provided in Supporting Information (Figure S8). As in the capillary geometry, the normalized SiV-DND/H_2_O and GeV-DND/H_2_O signals scale linearly with concentration under the glass-cell excitation conditions.

#### Glass-Cell Enrichment Experiments

Figure 5a shows the time evolution of the enrichment factor in the glass-cell experiment under the standard configuration, where counter-propagating pump beams (2.33 eV, 100 mW × 2) and a SiV-ZPL-resonant manipulation beam (1.68 eV, 100 mW) are applied. After 250 min, the enrichment factor increases to ≈1.5, demonstrating selective enrichment of SiV-DNDs consistent with an SRF-assisted mechanism.

**Figure 5:**
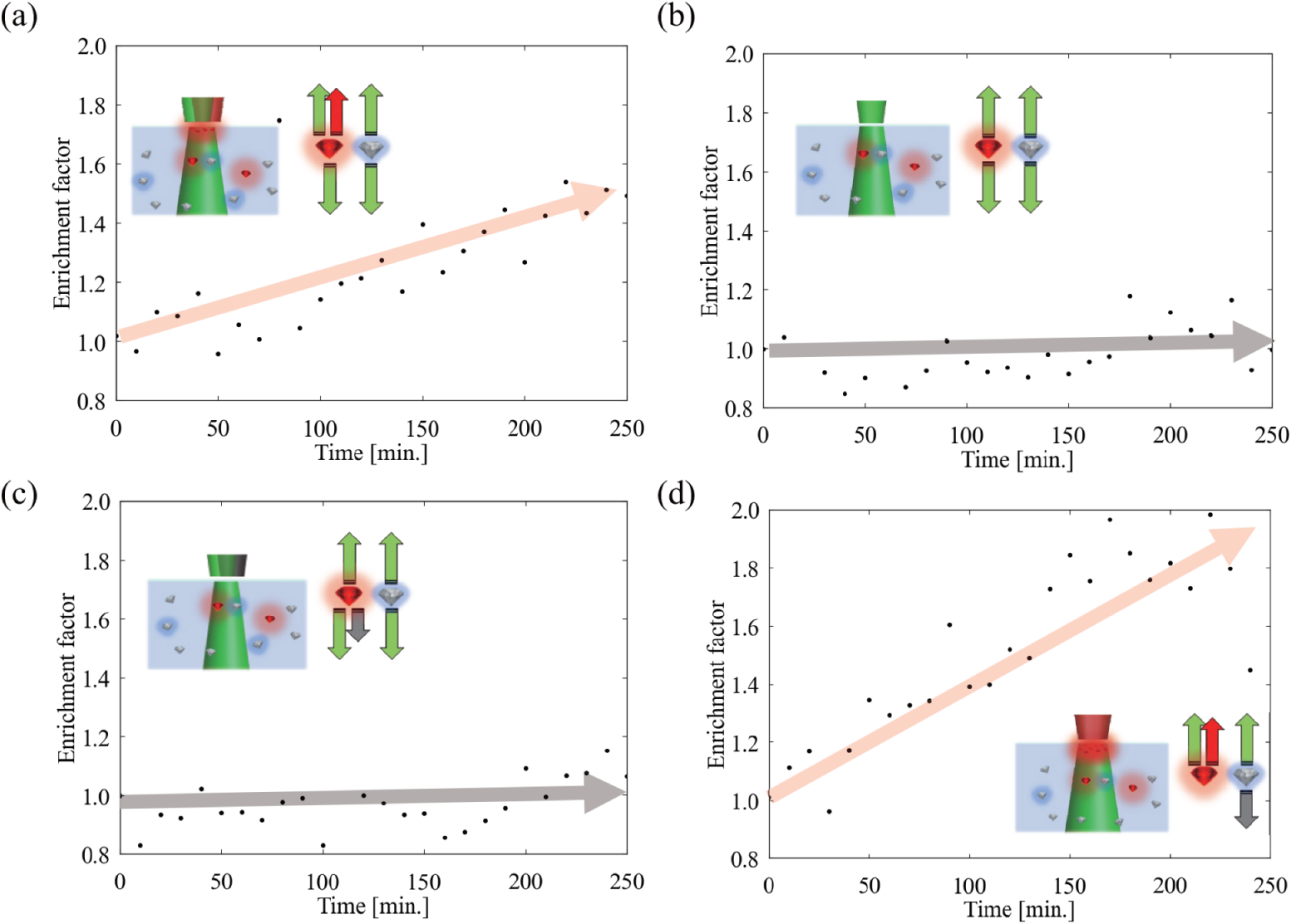
Time evolution of the enrichment factor in the glass-cell experiment. The enrichment factor is defined as *E*(*t*) = [(*I*_SiV_/*I*_GeV_)­(*t*)]/[(*I*_SiV_/*I*_GeV_)­(*t* = 0)], using the background-subtracted, Raman-normalized ZPL intensities. (a) Standard configuration: counter-propagating pump beams (2.33 eV, 100 mW × 2) and a SiV-resonant manipulation beam (1.68 eV, 100 mW) from the top yield selective enrichment (*E* > 1). (b) Pump-only control and (c) detuned control (manipulation at 1.58 eV) show no appreciable selectivity, with *E*(*t*) remaining near 1. (d) Enhanced configuration: pump applied only from the bottom (2.33 eV, 200 mW) so that absorption forces and SRF act constructively, resulting in a larger *E*(*t*). The solid arrows are guides to the eye.

To clarify the origin of this selectivity, we performed two control experiments. With the manipulation beam turned off, the enrichment factor remains close to 1 (Figure 5b). Similarly, when the manipulation photon energy is detuned to 1.58 eV, the enrichment factor stays near 1 (Figure 5c). Thus, selective enrichment occurs only when the manipulation laser is resonant with the SiV-ZPL, strongly suggesting that the mechanism is tied to stimulated emission in the SiV centers. In addition, we performed control measurements to assess possible photoionization (charge-state conversion) effects; these results show no evidence that ionization-driven changes account for the observed selectivity (see Supporting Information, Figure S10).

To further enhance the net force on SiV-DNDs, we modified the configuration so that SRF and absorption force add constructively: only the manipulation laser (1.68 eV, 100 mW) was focused from the top, while the pump laser (2.33 eV, 200 mW) was applied from the bottom. In this case, the absorption force on SiV-DNDs is no longer canceled and acts in the same direction as SRF; moreover, stimulated-emission-assisted rapid relaxation is expected to reduce saturation effects. In contrast, for GeV-DNDs, the manipulation beam is far detuned from the GeV-ZPL. Therefore, GeV-DNDs cannot benefit from a stimulated-emission-assisted rapid relaxation pathway, and any absorption force is expected to be limited by absorption saturation. Consequently, the absorption force on GeV-DNDs remains much smaller than that on SiV-DNDs and is therefore expected to contribute only marginally to the enrichment under this condition. As shown in Figure 5d, after 250  min, the enrichment factor reaches ≈1.9. Compared with the standard configuration, the enrichment is enhanced by ≈1.3, demonstrating that the cooperative action of SRF and absorption-based forces substantially improves selective enrichment.

Pump-only and detuned-manipulation configurations (Figure 5b and c) yield essentially no selectivity, with the enrichment factor remaining close to unity throughout the measurement. This indicates that any nonselective processes, such as adsorption to the glass interface or weak convection, affect the two species nearly equally and therefore do not change their ratio. These two conditions differ in the total optical load: the pump-only case contains only the counter-propagating pump beams (2.33 eV, 100 mW × 2), whereas the detuned case includes an additional 100 mW manipulation beam at 1.58 eV. Because this manipulation beam is far from the SiV-ZPL, it contributes only nonresonant gradient and scattering forces, yet still does not produce a measurable deviation of the enrichment factor from 1.

In contrast, when the manipulation beam is tuned to the SiV-ZPL (Figure 5a and d), the nonresonant optical forces (gradient + off-resonant scattering) acting on both species remain comparable to those in the detuned configuration because the manipulation power is the same. The key difference is that, in the resonant case, the manipulation beam now drives stimulated emission in SiV-DNDs and generates an SRF contribution that is spectrally locked to the SiV transition. Therefore, the comparison between the detuned and resonant configurations isolates the SRF-assisted component added on top of comparable nonresonant optical forces and slow adsorption or convection.

The glass-cell configuration exhibited higher reproducibility than the capillary geometry. We repeated the measurement on several independently prepared cells and evaluated the enrichment factor *E* using the same definition and the same ± 5% (1σ) success criterion as in the capillary measurements. In the standard configuration, we performed six runs under nominally identical conditions, yielding enrichment factors in the range *E* = 1.13–1.54. All six runs exceeded the 1σ threshold and were therefore categorized as successful. The distribution of *E* values obtained in the glass-cell experiments is summarized in Figure S9. The residual variation among runs is attributed primarily to the sensitivity of the experiment to (i) the mutual alignment of the counter-propagating pump beams and (ii) micrometer-scale adjustments of the focal position relative to the upper glass interface. Nevertheless, even in runs where the magnitude of enrichment varied, the qualitative behavior was fully consistent: SiV-DNDs exhibited clear enrichment under SiV-resonant conditions, whereas no selective redistribution occurred in the pump-only or detuned configurations. This reproducibility in a well-defined focusing geometry confirms the robustness of the SRF-assisted mechanism.

### Optical-Force Modeling and Brownian-Dynamics Simulations

To elucidate the physical origin of the selective enrichment observed in the glass-cell experiment, we performed optical force calculations and Brownian-dynamics simulations using the experimentally relevant beam geometry and SiV-center parameters (details are provided in Materials and Methods and the Supporting Information).

Figure 6a shows the calculated *y* component of the optical force field for the standard configuration. In the standard case (gradient force + SRF), the net force acting on SiV-DNDs reaches a peak value of ∼1.4 fN. In the enhanced case (gradient + SRF + absorption force; Figures S1–S2a), where SRF and the absorption force act in the same direction, the net force on SiV-DNDs increases to a peak value of ∼1.6 fN. In the gradient-only case, in which SRF is absent (Figures S1–S2c), the net force reaches a peak value of ∼1.1 fN. Because the manipulation-laser photon energy in the glass-cell experiments is far detuned from the GeV-ZPL, GeV-DNDs are expected to experience a nonresonant force of comparable magnitude to this gradient-only case. We therefore use the gradient-only configuration as a proxy for the GeV-DND response. With this interpretation, the net forces acting on SiV-DNDs in the standard and enhanced configurations are approximately 1.3 and 1.5 times larger, respectively, than that acting on the GeV-DND control. To provide an intuitive measure of the force magnitude relative to thermal diffusion, we estimated the drift–diffusion balance using the Stokes–Einstein relation and the corresponding Péclet number (Supporting Information, Section S3). Using the peak force in the standard configuration (∼1.4 fN), we obtain *P*e = O(1) on the micrometer length scale relevant to the focused-beam geometry, indicating that SRF-assisted drift can compete with thermal diffusion in the focal region.

**Figure 6:**
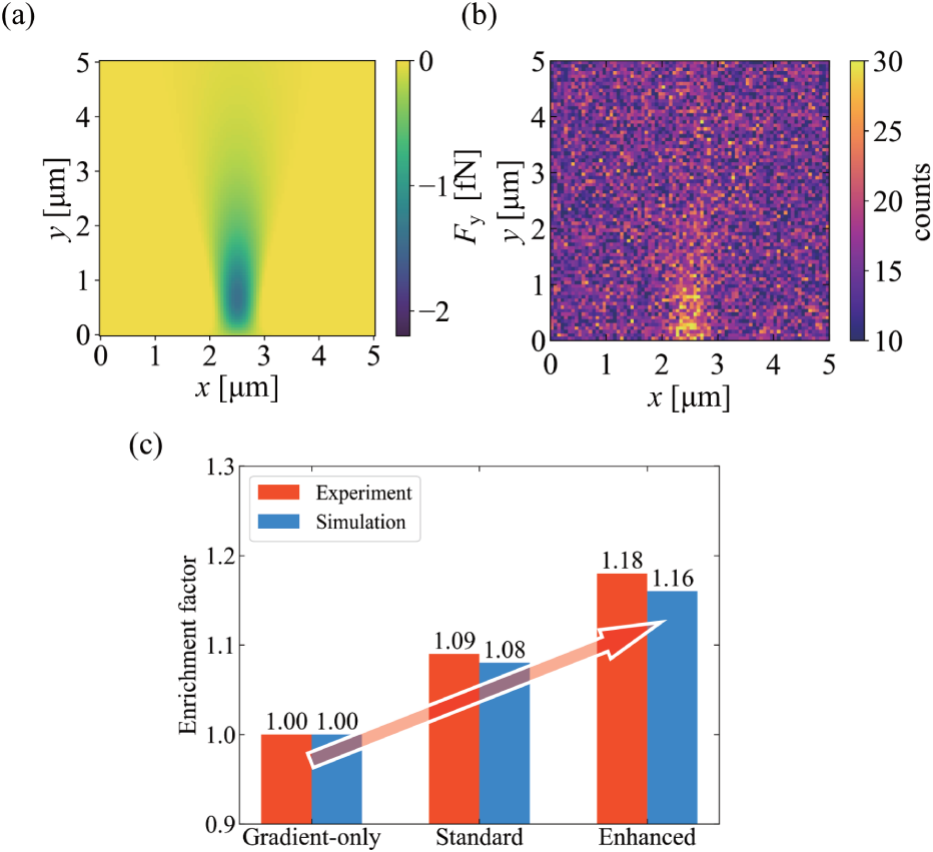
(a) Calculated *y*-component of the optical force on SiV-DNDs for the standard configuration. (b) Final particle density from Brownian-dynamics simulations, showing enrichment near the focal region. (c) Simulated enrichment factors for each configuration (Gradient only, Standard, Enhanced), in qualitative agreement with experiment.

To examine whether SRF can promote the enrichment of SiV-DNDs in water, we performed two-dimensional Brownian-dynamics simulations using the optical-force fields in Figure 6a. Figure 6b shows the final particle-density distribution under the standard configuration, revealing an increased density near the focal region.

To quantify enrichment in a manner consistent with the experimental definition based on the SiV-to-control ratio, we evaluate a simulated enrichment factor *E* (see Materials and Methods for the definition). As summarized in Figure 6c, the resulting enrichment factors are approximately 1.00, 1.08, and 1.16 for the gradient-only, standard, and enhanced cases, respectively, in qualitative agreement with the experimental values of approximately 1.00, 1.09, and 1.18.

We emphasize that this agreement should not be interpreted as a fully quantitative fit. In principle, the absolute values of the simulated enrichment factors can depend on processes such as the adsorption of particles onto the glass wall, where they are pushed by the optical forces, as well as on parameters including transition dipole moments and radiative and nonradiative decay rates. In addition, our simulations describe only a two-dimensional slice obtained by projecting the underlying three-dimensional force field near the glass interface. Rather, Figure 6c demonstrates that our Brownian-dynamics model successfully captures the trend of enhanced enrichment when an SRF contribution and an additional absorption contribution that is less affected by saturation are added on top of the gradient forces. The on/off and resonant/off-resonant comparisons already isolate the dissipative SRF contribution sufficiently for our purpose.

## Conclusion

We have shown that optical manipulation using SRF can provide a promising route to selective transport for group-IV-vacancy nanodiamonds in liquid. In a glass capillary with counter-propagating pump beams and a manipulation beam tuned to the SiV-ZPL, SiV-DNDs are enriched near the manipulation-laser input side and depleted several millimeters downstream, whereas the GeV-DNDs’ concentration remains essentially unchanged. These observations show that dissipative optical forces acting selectively on SiV-DNDs can measurably bias diffusion over millimeter-length scales and enable long-range, color-center-selective transport of SiV-DNDs in a capillary at a performance level that merits consideration for scalable microfluidic implementation. In the capillary system, we observe a 50% ( 1.5×) enrichment over 7 h. If this per-stage gain can be maintained and accumulated in the collected fraction, a back-of-the-envelope extrapolation suggests that order-of-10^2^ enrichment could be achieved on a time scale of a few days. Such cumulative enrichment could provide a practical route to increasing the amount of SiV-DNDs usable for downstream applications. In particular, implementing SRF-based sorting in a continuous-flow fractionation scheme (e.g., multistage microfluidic cascades with recirculation) should enable higher enrichment with shorter processing times and improved throughput.

To establish that SRF is the mechanism underlying the observed selective transport, we have performed complementary glass-cell experiments under a well-defined focusing geometry and directly monitored the local concentrations of SiV-DNDs and GeV-DNDs. By varying the resonance condition, we separated the contributions of selective dissipative forces from those of gradient forces. Optical-force calculations based on a four-level model of the SiV^–^ center, combined with Brownian-dynamics simulations, reproduce the experimentally observed trends in the enrichment factors and show that including an SRF contribution can raise the effective optical force above the gradient-only case while remaining compatible with nanoscale particles in aqueous media.

These findings indicate that optical forces that include an SRF contribution are suitable mechanisms for the long-range, selective manipulation of F-NDs and provide quantitative design guidelines for engineering SRFs that favor a targeted color center. More broadly, the combination of capillary-scale experiments, microscopic control in a glass cell, and force-based modeling outlined here provides a general framework for designing wavelength-selective optical sorting schemes in capillaries and microfluidic platforms, and it can be extended to other fluorescent nanoparticles and multicomponent colloidal systems.

## Materials and Methods

### Sample Preparation

We used water dispersions of SiV-DNDs and GeV-DNDs. Both types of DNDs were synthesized, purified, and post-treated by gas-phase oxidation at 570^◦^C following previously reported methods,^34,35^ yielding SiV-DNDs and GeV-DNDs with mean particle diameters of ∼10 nm. The resulting DNDs were then modified with polyglycerol (PG) and dispersed in water by tuning the ionic strength, following a reported procedure.^66^ Details of these procedures are provided in the Supporting Information, Sec. S4.

In this work, we focus on SiV-DNDs as the target particles for selective enrichment. However, undoped DNDs do not exhibit spectrally sharp features that would allow their local concentration to be quantified reliably from the photoluminescence (PL) spectrum. To circumvent this limitation, we instead employ GeV-DNDs as spectrally distinguishable reference F-NDs. GeV centers provide a narrow ZPL at a distinct energy and remain far off-resonant with respect to the SiV manipulation scheme used here. Under these conditions, the GeV-DNDs act as an experimentally convenient proxy for an off-resonant DND population, whose concentration can be monitored independently via its own ZPL. For the capillary experiments, we used a 0.5 wt % mixed dispersion of SiV-DNDs and GeV-DNDs in water. For the glass-cell experiments, we used a 0.05 wt % mixed dispersion of the same components.

The SiV-DNDs used in this study are expected to exhibit single color-center occupancy. To assess this expectation, we place an upper bound on the number fraction of SiV-DNDs in the mixed dispersion using independent indicators. Based on (i) a reported NV^–^ concentration in comparable 10 nm DNDs^67^ and (ii) the relative SiV contribution in the ensemble PL spectrum (Figure 2a), we estimate that the SiV-DND number fraction is at most on the order of ∼10^–3^. At such a low fraction, the probability that a 10 nm DND hosts two or more SiV centers is expected to be negligibly small; therefore, the SiV-DNDs studied here are reasonably regarded as having single color-center occupancy. A detailed description is provided in the Supporting Information, Sec. S5.

### Capillary Experiment

#### Capillary Preparation

Glass capillaries with an outer diameter of 1 mm and an inner diameter of 20 μm were filled with a 0.5 wt % mixed SiV-DND/GeV-DND aqueous dispersion and sealed at both ends with grease to prevent evaporation during long-term irradiation.

#### Optical Setup and Irradiation Conditions

A continuous-wave (CW) diode-pumped solid-state laser at 2.33 eV (MGL-N-532 nm-5W-A, Good Beam, CivilLaser) served as the pump laser. The pump beam was split into two beams (1.5 W each) and injected from opposite ends of the capillary. This counter-propagating geometry provided excitation while largely canceling the net axial absorption force.

A second CW laser (Matisse C, Spectra-Physics), tunable from 1.2 to 1.8 eV with an output power of 500 mW and a linewidth below 20 MHz, was launched from one end of the capillary as the manipulation beam. For SRF measurements, the manipulation photon energy was set to 1.68 eV, resonant with the SiV-ZPL. In control experiments, the manipulation beam was either blocked or detuned from the SiV-ZPL (e.g., to 1.58 eV) at nearly the same optical power, thereby suppressing SRF.

For the capillary configuration, the pump and manipulation beams were delivered as weakly focused beams with an effective NA of ∼0.02 (estimated from the beam diameter and the focal length of the lens). This design enables spatially resolved observation of accumulation and depletion within the capillary.

#### PL Measurement Protocol and Observation Positions

PL spectra were acquired using a 2.33 eV CW laser (LCX-532S, Oxxius) and a spectrometer (150 gr./mm) equipped with a CCD detector. Strong scattered light from the pump and manipulation beams prevented simultaneous PL measurements during irradiation. We therefore employed an alternating protocol (Figure 1b), where 5 min of irradiation with the pump and manipulation lasers was followed by a 20 s PL readout with both lasers blocked by shutters.

PL spectra were recorded at two observation positions along the capillary: position  #1 located 1 mm from the manipulation-beam input end, and position  #2 located 3 mm from the same end. Different capillaries prepared under identical conditions were used for the measurements at each position.

### Glass-Cell Experiment

#### Cell Preparation and Thickness

A rectangular well was prepared on a glass slide using a vacuum-grease frame and filled with a 0.05 wt % mixed SiV-DND/GeV-DND aqueous dispersion. A cover glass was placed on top and sealed with grease to form a closed cell. The cell thickness was estimated from the focal offset between the upper and lower glass interfaces and was typically 50–100 μm.

#### Optical Configuration

The glass cell was placed on an inverted microscope. A CW pump laser at 2.33 eV was split into two beams and focused onto the sample from the top and bottom through NA 0.4 objectives (100 mW each). This counter-propagating geometry provided excitation while largely canceling the absorption force along the vertical direction.

A CW manipulation laser at 1.68 eV (SiV-resonant) was focused from the top through the same objective (100 mW) to generate SRF on SiV-DNDs. Because GeV-DNDs are detuned by ∼0.4 eV at this photon energy, they experience only nonresonant optical forces under these conditions. The focal position for both excitation and detection was set at the upper glass interface to preferentially probe the near-surface region where SRF-assisted accumulation occurs.

#### PL Measurement Protocol

The pump and manipulation beams were applied continuously. Every 10 min, the manipulation beam was blocked for 1 s while keeping the pump on, during which a PL spectrum was recorded [Figure 4b].

### Optical-Force Calculations and Brownian-Dynamics Simulations

To quantify the forces underlying the glass-cell experiments, we calculated the time-averaged optical force ⟨**F­(**ω**)**⟩ acting on a dielectric particle from the electromagnetic field and polarization distributions as

2 ⟨F(ω)⟩=12Re∫dr{∇E*(r,ω)}·P(r,ω)

where **E**(**r**,ω) is the electric field and **P**(**r**,ω) is the induced polarization.^56^ In the resonant polarization calculations, the SiV-DNDs were modeled as an ensemble of randomly oriented four-level systems, and SiV center parameters reported previously for NDs were used (see Supporting Information, Sec. S1).^65^

The coordinate system is defined as in Figure 4a: the focal point is located at *F* = (*x,y*) = (2.5 μm, 0), the manipulation beam propagates along the +*y* direction, and the pump beam propagates either from both +*y* and −*y* (standard configuration) or from −*y* only (enhanced configuration). The optical-force fields used in the main text (Figure 6) were computed for these beam geometries.

#### Brownian-Dynamics Simulations

To examine whether the calculated force fields promote the enrichment of SiV-DNDs in water, we performed two-dimensional Brownian-dynamics simulations using the optical-force fields shown in Figure 6a. The simulations were carried out in a 5 μm × 5 μm domain with reflecting boundary conditions. We used a particle diameter of 15 nm and a diffusion coefficient corresponding to water at room temperature, and chose the total integration time to match the 60 min irradiation time. In each run, we propagated 1× 10^5^ particles that were initially distributed uniformly over the domain. Their motion was governed by the position-dependent optical-force fields together with thermal noise corresponding to room-temperature diffusion. This two-dimensional model describes a thin layer near the upper glass interface where the optical forces are largest (see Supporting Information, Sec. S2).

#### Definition of the Simulated Enrichment Factor

To quantify enrichment in a manner consistent with the experimental definition based on the SiV-to-control ratio, we define a simulated enrichment factor *E* as

$$E=\frac{(N_{\mathrm{SiV}}/N_{\mathrm{GeV}}{)}_{\mathrm{final}}}{(N_{\mathrm{SiV}}/N_{\mathrm{GeV}}{)}_{\mathrm{initial}}}$$

Here, *N*_SiV_ and *N*_GeV_ denote the numbers of particles in the region 2.0 < *x* < 3.0 μm and 0 < *y* < 0.1 μm. In the simulations, “GeV” refers to the gradient-only (GeV-proxy) configuration, which approximates the nonresonant response under the present glass-cell conditions. Because both species are initially uniform in the simulations, (*N*_SiV_/*N*_GeV_)_initial_ = 1, so that *E* reduces to *E* = *N*_SiV,final_/*N*_GeV,final_.

## Supplementary Material



## Notes

Details of optical-force calculations, Brownian-dynamics simulations, drift–diffusion analysis based on the Stokes–Einstein relation and Péclet number, preparation of SiV-/GeV-DND water dispersion, estimation of SiV-center occupancy, experimental stability and temperature analysis, concentration calibration, reproducibility tests, and control experiments excluding photoionization (PDF)
