# Supplementary material for: Selective Enrichment of Fluorescent Nanodiamonds by Stimulated Recoil Forces
Source: ACS Nano. 2026 Mar 26;20(13):10617–27. doi: 10.1021/acsnano.5c22759 (PMC13063815; doi:10.1021/acsnano.5c22759)
Supplement: Supplementary file 1 [file nn5c22759_si_001.pdf]

Selective Enrichment of Fluorescent Nanodiamonds by Stimulated Recoil Forces

Yoshiki Saito,[†] Takao Horai,[†] Yoshiki Umekawa,[†] Ryosuke Shimono,[†] Yoshihiro Tomoi,[†] Takuya Matsuda,[†] Yuto Makino,[‡,†] Yosuke Minowa,[¶] Hajime Ishihara,[§,‖,⊥] and Masaaki Ashida[*,†]

†*Graduate School of Engineering Science, The University of Osaka, 1-3 Machikaneyama-cho, Toyonaka, Osaka 560-8531, Japan*

‡*Daicel Corporation, 1239 Shinzaike, Aboshi-ku, Himeji, Hyogo 671-1283, Japan*

¶*Department of Physics, Kyoto University, Kitashirakawa-Oiwake-cho, Sakyo-ku, Kyoto 606-8501, Japan*

§*Research Organization of Science and Technology, Ritsumeikan University, 1-1-1, Nojihigashi, Kusatsu, Shiga 525-8577, Japan*

‖*SANKEN, The University of Osaka, 8-1 Mihogaoka, Ibaraki, Osaka 567-0047, Japan*

⊥*Ritsumeikan Semiconductor Application Research Center (RISA), Ritsumeikan University, 1-1-1 Nojihigashi, Kusatsu, Shiga 525-8577, Japan*

E-mail: ashida@mp.es.osaka-u.ac.jp

Supporting information

Calculation

S1. Method for Calculating Optical Forces

To evaluate the optical forces applied to SiV-DNDs and their transport distances for selective transport utilizing SRF, we employed the general expression for the time-averaged Lorentz force[1].

$$\langle \boldsymbol{F}(\omega)\rangle = \frac{1}{2}\mathrm{Re}\left[\int \mathrm{d}\boldsymbol{r}\sum_{\omega}\{\nabla \boldsymbol{E}^{*}(\boldsymbol{r},\omega)\}\cdot \boldsymbol{P}(\boldsymbol{r},\omega)\right],$$

where $\boldsymbol{E}$ and $\boldsymbol{P}$ represent the electric field and polarization, respectively, with the latter comprising both resonant and non-resonant (background) polarization. ω denotes the angular frequency of the incident light, and r represents the particle position. The resonant-induced polarization can be determined by solving the density matrix equation for the material system.[2] The equation of motion for the density matrix, including phenomenological relaxation constants, is given by

$$i\hbar\frac{\partial\rho}{\partial t} = [H,\rho] - \Gamma\rho,$$

where $\hbar = h/2\pi$ (h is Planck's constant), and ρ , Γ , and H represent the density operator, phenomenological relaxation constants, and total system Hamiltonian $H = H_0 + V$, respectively. H_0 is the unperturbed component, and V is the light-matter interaction energy, given by $V = -\mu\cdot E(r,t)$, where μ is the dipole moment operator with diagonal elements representing permanent dipole moments set to zero. For the electric field, considering the angular frequencies ω_1 and ω_2 of the pump laser and the manipulation laser:

$$E(\boldsymbol{r},t) = E_1(\boldsymbol{r},\omega_1)e^{-i\omega_1 t} + E_2(\boldsymbol{r},\omega_2)e^{-i\omega_2 t} + c.c.,$$

where E_1, E_2 represent the electric field amplitude of the pump laser and manipulation laser. While the electric field is typically described as the sum of incident and scattered fields, we neglect the scattered field's influence here as it is considered minimal. The diagonal and off-diagonal components of the density matrix equation of motion are expressed as:

$$\frac{\partial\rho_{jk}}{\partial t} = -\frac{i}{\hbar}[V,\rho]_{jk} - \left(i\Gamma_{jk} + \gamma_{jk}\right)\rho_{jk}$$

$$\frac{\partial\rho_{ll}}{\partial t} = -\frac{i}{\hbar}[V,\rho]_{ll} - \Sigma_{m>l}\Gamma_{ml}\rho_{mm} - \Sigma_{m<l}\Gamma_{lm}\rho_{ll}$$

where Γ_{jk} and γ_{jk} are the population relaxation and phase relaxation constants between levels j and k, respectively. In this paper, we expand the off-diagonal components of the density matrix in a Fourier series:

$$\rho_{jk}(t) = \Sigma_{n,m}\rho_{jk}^{(n,m)}\, e^{-i(n\omega_1+m\omega_2)t}$$

where j, k, n, and m are integers, and only the lowest order is considered for diagonal components. By solving these two equations of motion, we can determine each component of the density matrix and calculate the expectation value of the resonant-induced dipole moment $\mu_{\mathrm{res}} = \mathrm{Tr}[\rho\mu]$. Here, the resonant polarization scales linearly with the number of resonant color centers contained in a single ND. In the

present calculations, we assume a single SiV center per SiV-DND; the basis for this single-center assumption is given in the Materials and Methods and Section S5. [3]

The background component of the dipole moment μ_β can be calculated from the Clausius-Mosotti relation:

$$\overrightarrow{\mu_b} = \alpha_{\mathrm{CMRR}}\, \boldsymbol{E}(\boldsymbol{r}, \omega)$$

$$\alpha_{\mathrm{CMRR}} = \alpha_{\mathrm{CM}} \left(1 - ik^3 \frac{\alpha_{\mathrm{CM}}}{6\pi\epsilon_0\epsilon_2}\right)^{-1}$$

$$\alpha_{\mathrm{CM}} = 4\pi a^3 \epsilon_0 \epsilon_2 \frac{m-1}{m+2}$$

where ϵ_0, ϵ_1, and ϵ_2 are the permittivities of vacuum, target particle, and medium, respectively, and m = ϵ_0/ϵ_2. The permittivity of water was set to ϵ_2 = 1.8. Additionally, k is the wavenumber of the incident light, and a is the particle radius. Given these equations, we can calculate polarization by providing the electric field, thereby enabling us to determine the optical force.

Calculation Model

Figure S1-1(a) shows the models of SiV-DND and GeV-DND used in the calculations. Regarding the resonant polarization of SiV-DND, considering vibrational levels, we assumed that light absorption occurs between levels 1 and 2, and levels 1 and 3, while spontaneous emission occurs between levels 1 and 2. We set $\hbar\Omega_{41}$ = 1.71 eV, $\hbar\Omega_{32}$ = 1.65 eV, and $\hbar\Omega_{31}$ = 1.68 eV. For the dipole moments between levels, we set μ_{31} = 14.3 Debye, corresponding to the zero-phonon transition.[4] Based on the Huang-Rhys factor, we set μ_{32} = 7.3 Debye and μ_{41} = 6.7 Debye. For phase relaxation constants between levels, we assumed γ_{ij} = 2 meV (where $i \neq j$ and i, j = 1, 2, 3, 4), considering room temperature in water. For population relaxation constants between levels, we set $\Gamma_{43} = \Gamma_{21}$ = 15 meV, as relaxation between vibrational levels occurs very rapidly.[5] Based on experimental fluorescence lifetime, we set $\Gamma_{32} = \Gamma_{31}$ = 1.2 μeV.[6]

We evaluated the position dependence of the optical forces acting on each DND under the present focusing geometry. The electric fields of the manipulation and pump beams were calculated for a fundamental Gaussian beam focused by an objective lens with a numerical aperture of NA = 0.4. We assumed that the manipulation laser propagates along the +*y* direction, while one of the pump lasers co-propagates along +y and the other pump laser counter-propagates along −*y*. Using the fifth-order correction formula for the electromagnetic field components of a Gaussian beam,[7] we obtained the intensity distribution around the focal region. On the basis of this intensity distribution, we calculated the position dependence of the optical forces acting on SiV-DNDs and GeV-DNDs on a two-dimensional grid within a 5 μm × 5 μm region centered at the focus. These force distributions were subsequently used as input for the Brownian-dynamics simulations described in the following section.

Figure S1-2 shows the calculated optical-force distributions. Panels (a), (b), and (c) correspond to the enhanced configuration, the standard configuration, and the gradient-only case, respectively, and display the x- and y-components of the force as a function of position in the 5 μm × 5 μm region. In all

three configurations, the net optical force tends to point toward (x, y) = (2.5 μm, 0), i.e., toward the center of the high-intensity region. Even in the gradient-only configuration, the total force reaches about 1.1 fN. In the enhanced configuration [Fig. S1-2(a)], the combined action of SRF and non-saturated absorption forces increases the maximum force to approximately 1.6 fN, whereas in the standard configuration [Fig. S1-2(b)] the maximum force is about 1.4 fN. These results confirm that SRF-assisted and absorption-based forces substantially enhance the net optical force compared with the gradient-only case.

Figure S1-1

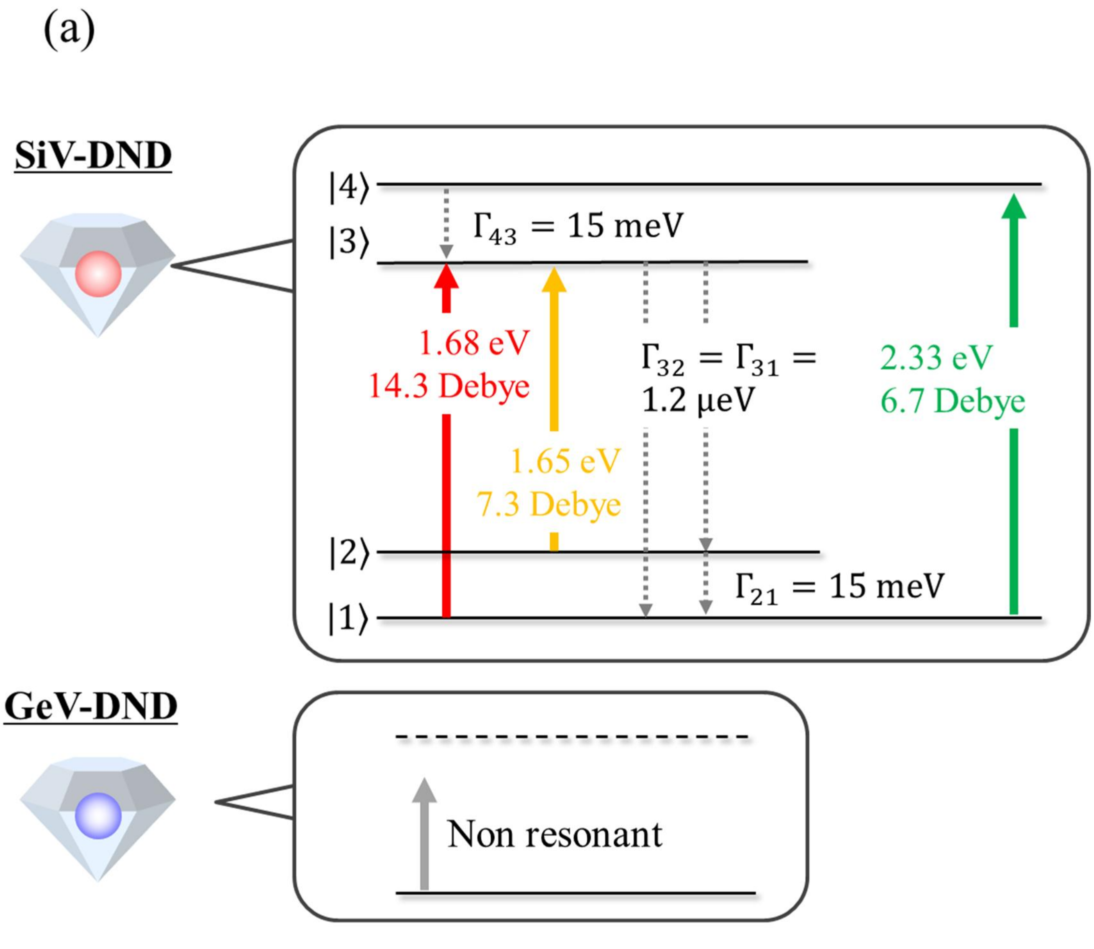

Energy-level schemes used in the optical-force calculations for (a) SiV-DNDs and (b) GeV-DNDs. The relevant optical transitions, pump and manipulation photon energies, and phenomenological relaxation rates are chosen so as to reproduce the experimentally observed fluorescence lifetimes.

Figure S1-2

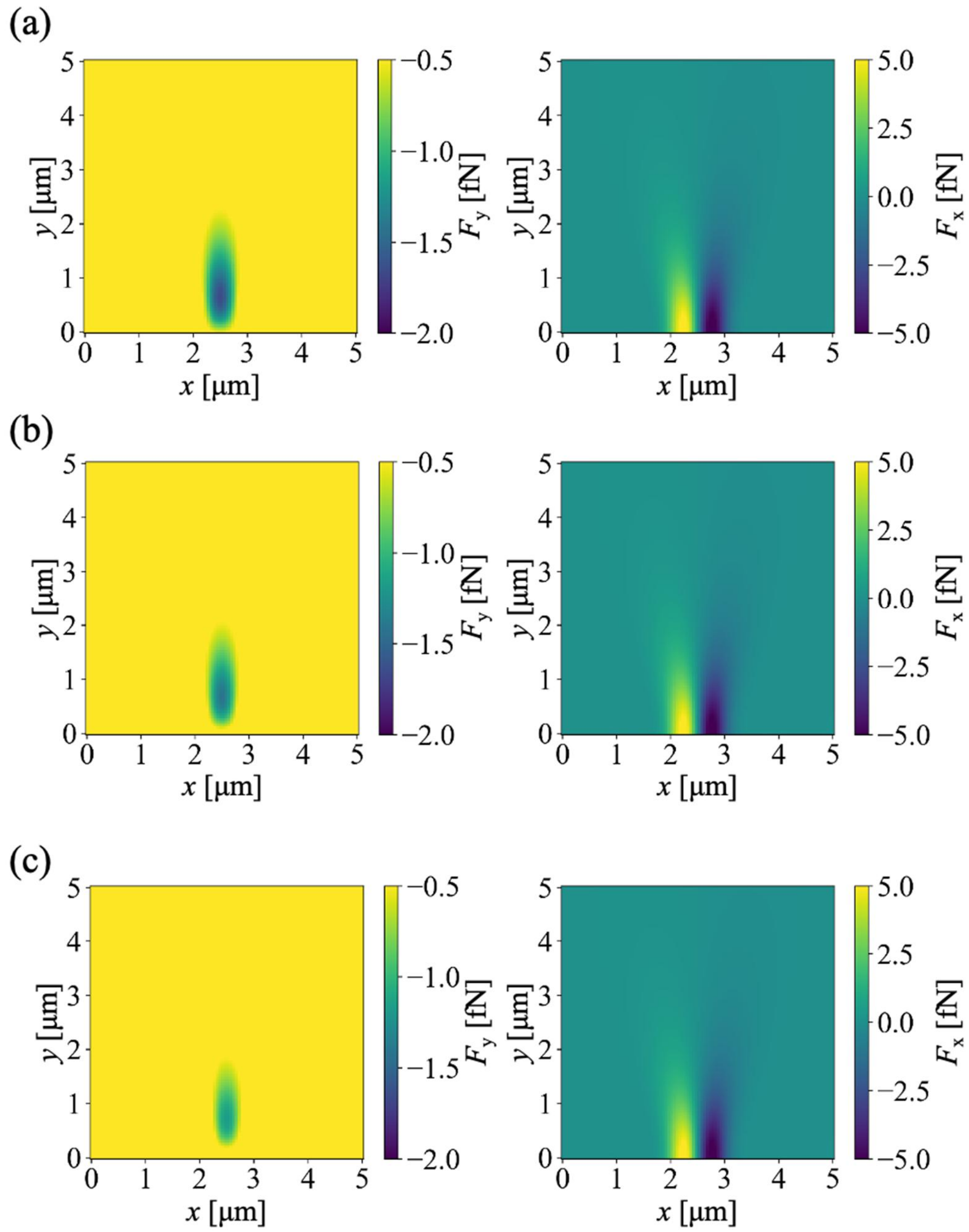

Calculated optical-force distributions acting on SiV-DNDs in the glass-cell geometry. The color maps show the spatial profiles of the force components for (a) Enhanced (gradient + SRF + absorption force) case, (b) Standard (Gradient + SRF) case, and (c) the gradient-force-only case. Inclusion of SRF and absorption force enhances the net optical force and biases particle motion toward the downstream side compared with the gradient-only case.

S2. Brownian-dynamics simulations with optical forces

To examine how the calculated optical forces bias the Brownian motion of nanodiamonds, we performed two-dimensional overdamped Brownian-dynamics simulations in a square domain, using the force fields obtained in "Glass cell experiment" (standard, enhanced, and gradient-only configurations). The motion of a nanodiamond in water is modeled by the overdamped Langevin equation in two dimensions,

$$\frac{d\boldsymbol{r}}{dt} = \mu_0\, \boldsymbol{F}(\boldsymbol{r}) + \sqrt{2D}\, \boldsymbol{\xi}(t),$$

where $\mathbf{r} = (x, y)$is the particle position in the plane, $\boldsymbol{F}(\boldsymbol{r}) = (F_x, F_y)$is the deterministic optical force, $\mu_0$is the mobility, D is the diffusion coefficient, and $\boldsymbol{\xi}(t)$ is a vector of independent Gaussian white noises with zero mean and unit variance.

The mobility and diffusion constant are related by the Stokes–Einstein relation,

$$\mu_0 = \frac{1}{\zeta}, D = \frac{k_B T}{\zeta}, \zeta = 6\pi\eta a,$$

with $k_B$the Boltzmann constant, T: the temperature, η: the viscosity of water, and a: the particle radius. In the simulations we use $T = 298$ K, $\eta = 0.890 \times 10^{-3}$ Pa $\cdot$ s, $a = 7.5$ nm, which yields a diffusion coefficient D consistent with 15-nm sized NDs in water and the corresponding mobility $\mu_0 = 1/\zeta$. The Langevin equation is integrated in discrete time using the Euler–Maruyama scheme,

$$\mathbf{r}(t + \Delta t) = \mathbf{r}(t) + \mu_0\, \mathbf{F}[\mathbf{r}(t)]\, \Delta t + \sqrt{2D\, \Delta t}\, \boldsymbol{\eta},$$

where $\boldsymbol{\eta}$ is a two-component vector whose elements are independent standard normal random numbers.

The deterministic optical force $\boldsymbol{F}(\boldsymbol{r})$ entering the Langevin equation is provided by the optical calculations described in "Glass cell experiment". For each configuration (standard, enhanced, gradient-only), we compute the force components $F_x(x, y)$and $F_y(x, y)$on a regular grid spanning a 5 μm × 5 μm region centered on the focal point $(x, y) = (2.5\ \mu\text{m}, 0\ \mu\text{m})$. The discrete force maps are then used as a lookup table: at each time step, the force at the current particle position is obtained by bilinear interpolation on this grid. The same Brownian-dynamics scheme is used for all configurations; only the force field $\boldsymbol{F}(\boldsymbol{r})$ is changed according to whether we include (i) gradient forces only, (ii) gradient + SRF, or (iii) gradient + SRF + non-saturated absorption forces (SRF-assisted case). In some simulations we also checked the effect of the increased viscosity near the glass surface using a first-order Brenner-type correction for the diffusion coefficient; this mainly reduces the overall diffusion rate but does not qualitatively change the enrichment behavior. For the enrichment factors reported in the main text, the dominant effect arises from the spatial variation of $\boldsymbol{F}(\boldsymbol{r})$.

From the simulated particle positions, we construct the steady-state density profile by binning the final positions r_i (t_{max}) into a two-dimensional histogram over the 5 μm × 5 μm box. The resulting density maps (analogous to those shown in Fig. 5(b) of the main text) clearly show an accumulation near the region where the optical force points toward the focal point. To quantify the enrichment, we define a small observation region corresponding to the experimentally probed area around (x, y) = (2.5 μm, 0) and

count the number of particles inside this region at $t = 0$ and at $t = t_{max}$. The enrichment factor E is defined as

$$E = N_{in}(t_{max})/N_{in}(0)$$

that is, the ratio of the final to the initial fraction of particles in the observation region. We evaluate E separately for the gradient-only, standard, and enhanced configurations. Figure S3 summarizes the simulation results for the three optical-force configurations. In the SRF + non-saturated absorption + gradient case [Fig. S2(a)], the local density at $(x, y) = (2.5\ \mu m, 0\ \mu m)$ is enhanced by a factor $E \approx 1.33$. In the SRF + gradient case [Fig. S2(b)], we obtain $E \approx 1.25$, while in the gradient-only case [Fig. S2(c)] the enrichment factor is $E \approx 1.15$. These results are consistent with the experimental trends and support the conclusion that SRF-assisted optical forces significantly enhance the local particle density compared with the gradient-only situation, thereby biasing Brownian motion and contributing to the observed selective enrichment.

Figure S2

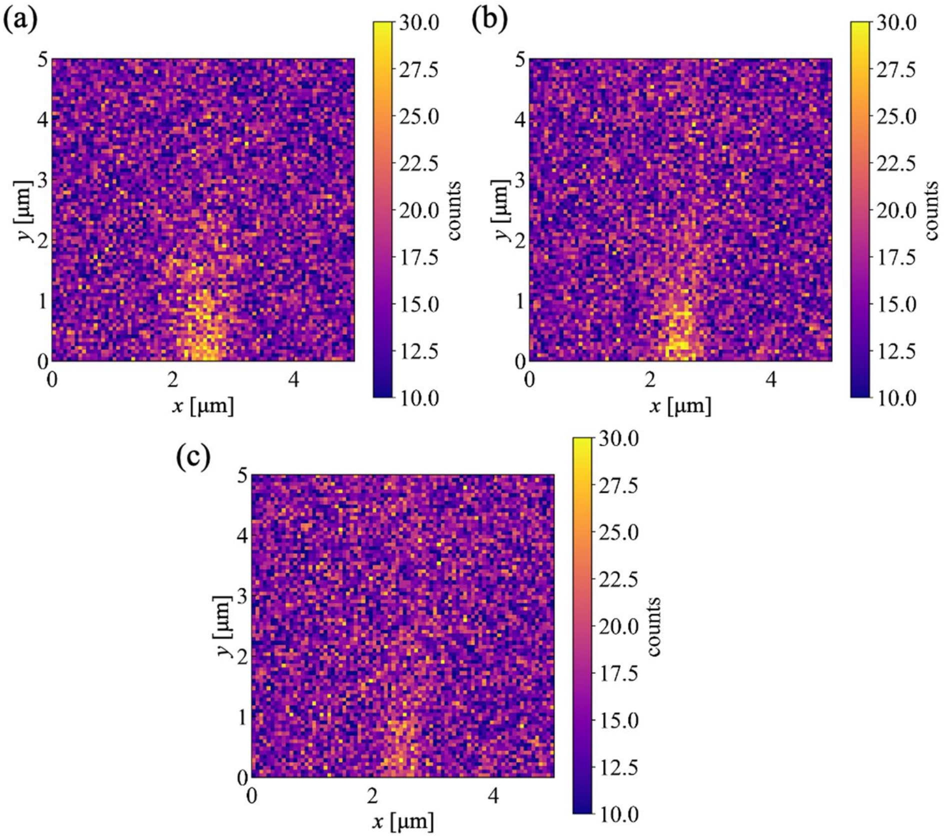

Brownian-dynamics simulations of nanodiamond transport in the presence of the calculated optical forces. Steady-state spatial distributions of particles near the glass interface for different force configurations ((a) SRF+ABS, (b) SRF, and (c) gradient-only). The simulations show that SRF-assisted forces are required to

obtain significant enrichment, whereas gradient forces alone produce only weak redistribution dominated by Brownian motion.

S3. Drift–diffusion balance: Stokes–Einstein estimate and Péclet number

This section provides a quantitative estimate of the drift–diffusion balance under our experimental conditions. We relate the optical force scale to the drift velocity via Stokes drag and to diffusion via the Stokes–Einstein relation, and we evaluate the corresponding Péclet number. This estimate supports that the stimulated-recoil-force (SRF)–assisted drift can compete with thermal diffusion in the focal region, consistent with the observed long-time redistribution/enrichment in both the microcapillary and glass-cell experiments.

S3.1 Definitions

For a particle of hydrodynamic radius, a in a fluid of viscosity η at temperature T, the Stokes drag coefficient is $\zeta = 6\pi\eta a$. The drift velocity under an optical force F is $v_d = F/\zeta$. The diffusion coefficient is given by the Stokes–Einstein relation, $D = k_{\mathrm{B}}T/\zeta$. The Péclet number over a characteristic length scale L is therefore $Pe = y_d L/D = FL/(k_B T)$. Notably, Pe is independent of η and a; η and a only enter y_d and D separately.

S3.2 Parameter values used

We use the peak net force obtained from our force-map calculation for the standard configuration, F_{peak} = 1.4 fN (see Fig. 6(a) in the main text). We take room temperature, T = 298 K, for which $k_B T$ = 4.11×10^{-21} J. For the separate estimates of v_d and D (not needed for Pe), we adopt the same effective particle size as used in our Brownian-dynamics simulations, i.e., a particle diameter of 15 nm. This choice was made in the simulations to be consistent with the diffusion coefficient of the particles in water and is used here for consistency with that simulation parameterization (see main text, Brownian-dynamics section). We use the viscosity of water at 298 K, η = 0.890 mPa · s. For the characteristic length scale L, we consider (i) the axial extent of the focal spot, L = 2 μm, and (ii) the length scale used in the Brownian-dynamics domain, L = 5 μm (see main text / SI, simulation setup). These two values provide a practical range for the force-affected region relevant to our experiments.

S3.3 Numerical estimates

Using $Pe = FL/(k_B T)$ with $F = 1.4 \times 10^{-15}$ N: For L = 2 μm: $Pe = (1.4 \times 10^{-15} \cdot 2 \times 10^{-6})/(4.11 \times 10^{-21})$=0.68. For L = 5 μm: $Pe = (1.4 \times 10^{-15} \cdot 5 \times 10^{-6})/(4.11 \times 10^{-21})$=1.7. Thus, Pe is of order unity on the micrometer length scales relevant to the focused-beam geometry. This indicates that SRF-assisted drift can compete with (and, depending on position within the force field, exceed) diffusion in the focal region. For completeness, using $\zeta = 6\pi\eta a$ with η = 0.000890 Pa · s and a = 7.5 $\times 10^{-9}$ m gives $\zeta = 1.26 \times 10^{-10}$ Ns/m, $y_d = F/\zeta = 1.1 \times 10^{-5}$ m/s = 11 μm/s, $D = k_{\mathrm{B}}T/\zeta = 3.3 \times 10^{-11}$ m^2/s = 33 μm^2/s. These values satisfy $Pe = y_d L/D$ and provide an intuitive scale for the drift speed relative to diffusion.

S3.4 Implication for the experiments

Because $Pe = O(1)$ in the focal region, the force-induced drift is not expected to be washed out immediately by diffusion. Instead, the spatially structured SRF field can bias particle transport locally, and this bias accumulates over long irradiation times, leading to the experimentally observed redistribution/enrichment along the microcapillary (e.g., increase around $z \sim 1$ mm and decrease around $z \sim 3$ mm in Fig. 3 and the corresponding behavior observed in the glass-cell configuration (Fig. 5). This drift–diffusion estimate therefore provides a quantitative consistency check supporting the interpretation of the experimental results.

Experiment

S4. Preparation of Fluorescent Nanodiamond Water Dispersions

Fluorescent nanodiamonds (F-NDs) and their water dispersions were prepared according to previously reported procedures [6, 8–11]. All synthesis, purification, and surface modification steps were carried out at Daicel Corporation.

S4.1 Detonation Synthesis of SiV-DNDs and GeV-DNDs

SiV-DNDs and GeV-DNDs were synthesized by a detonation method using cylindrical explosives that contained triphenylsilanol or tetraphenylgermane as heteroatom dopants. The explosives were composed of 2,4,6-trinitrotoluene (TNT), hexahydro-1,3,5-trinitro-1,3,5-triazine (RDX), and dopant at 59/40/1 wt% (TNT/RDX/dopant). 1010 g of each explosive was detonated in a CO_2 atmosphere to obtain detonation soot containing the doped nanodiamonds.

S4.2 Purification and Oxidative Post-Treatment

The detonation soot was purified by a sequence of acid treatment, alkaline treatment, and oxidative annealing.

First, the soot was treated in an acidic mixture of $H_2SO_4/HNO_3/H_2O$ = 9/76/15 (wt%) at 150 °C for 5 h. After the reaction mixture had cooled to 70 °C, deionized (DI) water was added, and the mixture was then reheated to 150 °C for an additional 5 h. The resulting precipitate was collected, rinsed thoroughly with DI water, and dried.

The dried precipitate was then treated in 8 M NaOH aqueous solution at 70 °C for 8 h. After the alkaline treatment, the precipitate was washed with DI water at room temperature and re-suspended in DI water. The pH of the suspension was adjusted to 3–4 by adding 1 M HCl, and the suspension was centrifuged at 8000*g* for 10 min (CR22G, Hitachi Koki). The collected precipitate was washed again with DI water and dried.

Finally, the purified samples were oxidized in an O_2/N_2 (4/96, vol%) atmosphere at 570 °C for 2 h. As reported previously [6, 8], the average particle sizes of the oxidized SiV- and GeV-NDs were evaluated to be approximately 10 nm from powder X-ray diffraction patterns and transmission electron microscopy images.

S4.3 Preparation of Polyglycerol-Modified Nanodiamond Dispersions

The SiV- and GeV-DND particles were modified with polyglycerol (PG), and the ionic strength of the suspending medium was increased to obtain individually dispersed DND colloids, following the established protocol for undoped DNDs. [11]

The DND powders were first dispersed in DI water at a concentration of 6.0 wt% and sonicated using an ultrasonic processor (UP400S, Hielscher Ultrasound Technology) at 400 W and 45% amplitude

for 180 min in the presence of ZrO_2 beads with a diameter of 30 µm. After sonication, the ZrO_2 beads were removed through decantation and then centrifuged at 20000*g* for 10 min, resulting in black dispersions.

The collected DND dispersion was dried at 105 °C for 2 h to obtain a solid residue. This residue (0.5 g) was then mixed with ethylene glycol (13.6 g), and glycidol (13.6 g) was added dropwise over 105 min while maintaining the temperature at 95−102 °C. The reaction mixture, which became a black dispersion, was stirred at 95–102 °C for an additional 4 h and then stirred overnight at room temperature.

The PG modification was quenched by slowly adding DI water (20 mL), which also degraded unreacted glycidol. The crude dispersion was diluted with DI water to a total volume of approximately 400 mL and concentrated to 20 mL by ultrafiltration membrane (Ultracel® membrane 100 kDa, Merck KGaA). This dilution/ultrafiltration cycle was repeated five times, yielding purified PG-modified SiV- and GeV-DNDs (PG-SiV-DNDs and PG-GeV-DNDs) as water dispersions.

Thermogravimetry–differential thermal analysis (TG–DTA) of the PG-SiV-DND and PG-GeV-DND samples (Fig. S4) indicated PG/DND mass ratios of approximately 1.6/1.0 for both types of F-NDs.

S4.4 Preparation of Mixed PG-SiV/GeV-DND Water Dispersions

These two dispersions were then mixed at a volume ratio of 5/1 (PG-SiV-DND/PG-GeV-DND) to obtain a mixed PG-SiV/GeV-DND water dispersion. Finally, NaCl was added to the mixed dispersion at a concentration of 0.01 M. This procedure yielded a 1 wt% mixed PG-SiV/GeV-DND water dispersion with electrolyte. For the optical manipulation experiments in the main text, the dispersion provided by Daicel was further diluted at The University of Osaka to 0.5 wt% for the capillary experiments and to 0.05 wt% for the glass-cell experiments.

Figure S4

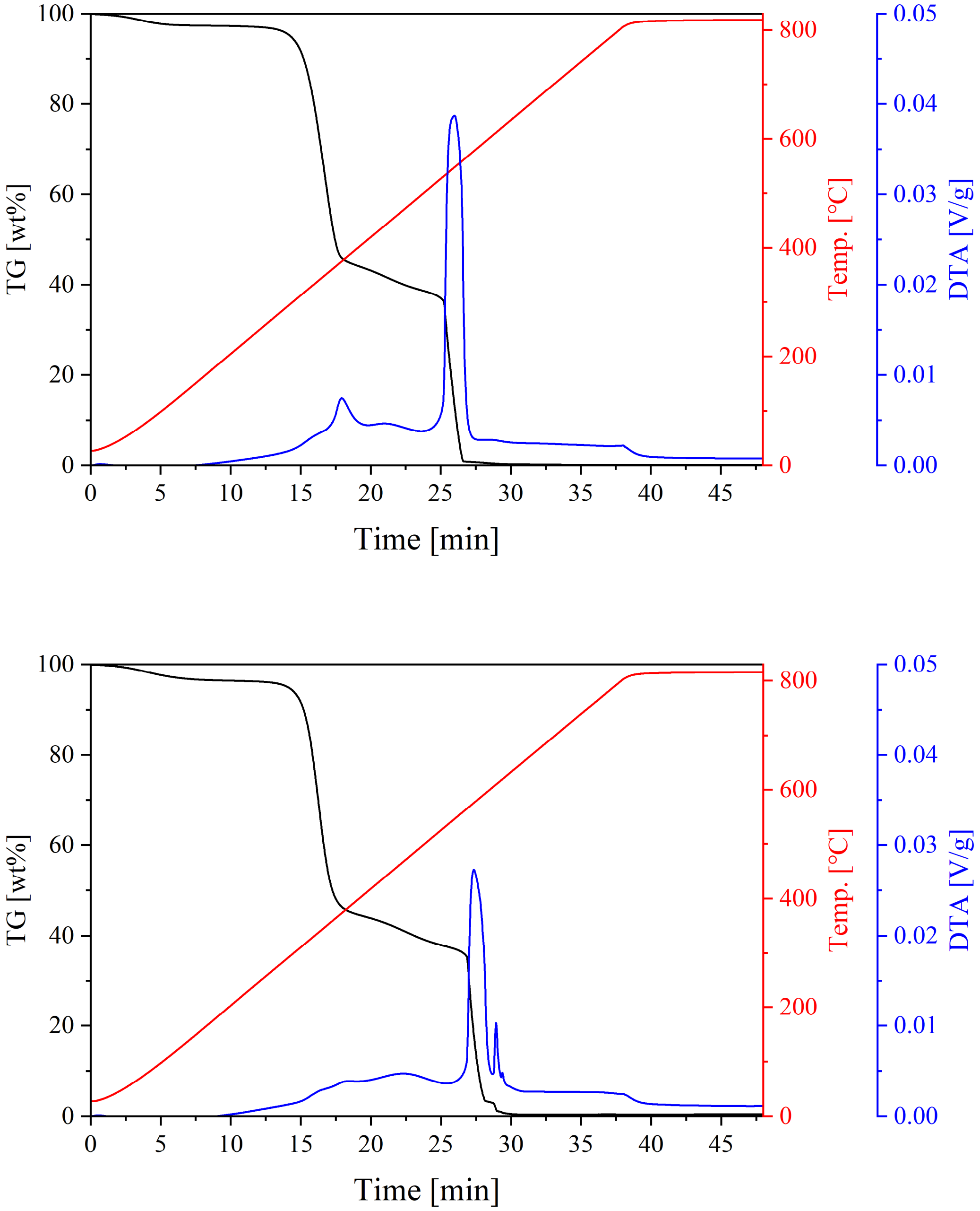

TG–DTA curves of PG–SiV–DND (top) and PG–GeV–DND (bottom) powders were obtained in air at 20 °C/min. The two samples show essentially the same thermal behavior. The initial few-percent weight loss during the first ~10 min arises from surface-adsorbed water.[12] Later mass losses are attributable to the PG layer and, at higher temperatures, to the nanodiamond core. [11, 12]

S5. Estimating the SiV-center occupancy and the negligible role of multi-center nanodiamonds

This section provides the quantitative rationale supporting the working approximation used in the main text that ~10 nm detonation nanodiamonds (DNDs) typically contain at most one optically active SiV center. While we cannot strictly exclude the existence of particles containing more than one SiV center, we show below that (i) the expected probability of two-or-more SiV centers is extremely small even under a conservative upper-bound estimate, and (ii) high incorporation/defect density is known to induce strong photoluminescence (PL) quenching of SiV-related emission, making multi-center particles unlikely to be optically dominant.

S5.1 Numerical extraction of I_{SiV}/I_{NV} from the measured PL spectra

We analyze the PL spectra shown in the main manuscript (Fig. 2(a)). The broadband background emission is attributed to a mixture of (i) NV^- emission, (ii) NV^0 emission, and (iii) other defect/surface-related luminescence. As a conservative working assumption, we take one half of the broadband background to originate from NV^- emission (the remaining half being NV^0 and other contributions). With this assumption, we define the following integration windows:

SiV-ZPL window: 1.6–1.7 eV

NV^- window: 1.5–1.9 eV

Baseline treatment: [polynomial baseline]

We then compute the integrated intensities as:

$$I_{SiV} = \int_{1.6}^{1.7} (I - baseline) \cdot d(eV)$$

$$I_{NV} = \int_{1.5}^{1.9} (baseline) \cdot d(eV)$$

Using the representative spectra in Fig. 2(a), we obtain a typical ratio:

$I_{SiV}/I_{NV} \sim 0.02$.

This ratio is used below only for an order-of-magnitude, conservative upper-bound estimate.

S5.2 Converting NV^- concentration to "per-particle occupancy" for 10 nm DNDs

We use the NV^- concentration in the SiV-DNDs reported by So et al. [3], which reports an NV^- density of approximately 0.35 ppm. Converting this concentration to the expected number of NV^- centers in a 10 nm-diameter nanodiamond yields an average occupancy of approximately: $\lambda_{NV} \sim 0.03$ per particle, i.e., about 3% of 10 nm particles contain one NV^- center (Poisson estimate: $P(\geq 1) = 1 - \exp(-0.03) \approx 0.03$).

S5.3 Conservative upper-bound estimate of SiV occupancy and Poisson bound on double occupancy

We now infer the mean occupancy of optically active SiV centers by combining the above quantities: $\lambda_{SiV} \sim \lambda_{NV} \times (I_{SiV}/I_{NV}) \sim 0.03 \times 0.02 = 6 \times 10^{-4}$. For simplicity, and to keep the estimate conservative, we round this to an upper bound: $\lambda_{SiV} \leq 1 \times 10^{-3}$, corresponding to roughly one SiV-containing DND per

~1000 particles. This estimate is intentionally conservative because the radiative dipole moment (oscillator strength) of SiV is reported to be larger than that of NV. Therefore, for the same center number, the SiV contribution would tend to appear stronger in PL; ignoring this difference can only overestimate λ_{SiV}, not underestimate it. Assuming a Poisson distribution for the number k of SiV centers per particle with mean λ= 0.001 (upper bound), we have:

$P(0) = \exp(-\lambda)$

$P(1) = \lambda \cdot \exp(-\lambda)$

$P(2) = (\lambda^2/2) \cdot \exp(-\lambda)$

Hence, the fraction of two-center particles among SiV-containing particles is: $P(2| \geq 1) = P(2)/(1 - P(0)) \approx \lambda/2 \approx 5 \times 10^{-4}$. That is, even under the conservative upper bound $\lambda = 0.001$, only ~0.05% (about 1 in 2000) of SiV-containing nanodiamonds would host two SiV centers. The absolute probability is $P(2) \approx 5 \times 10^{-7}$ (~1 in 2 million particles).

S5.4 Quenching at high incorporation densities further suppresses optical dominance of multi-center particles

Finally, high Si/defect incorporation is known to induce strong quenching of SiV-related photoluminescence. Bolshakov et al. reported that the SiV PL intensity increases up to an optimal Si incorporation (near SiH_4/CH_4 ~0.6%) and then exhibits rapid PL quenching at higher Si incorporation (see their Fig. 1 inset).[13] This behavior supports the general principle that higher incorporation/defect density opens efficient nonradiative pathways. Therefore, even if a minute fraction of nanodiamonds contains more than one SiV center, such particles are not expected to become proportionally brighter and are unlikely to dominate the experimentally observed SiV signal and the enrichment metrics. These quantitative considerations (Sections S5.1–S5.4) justify treating multi-center SiV nanodiamonds as negligible in the context of the present ensemble enrichment experiments.

S6. Stability and Measurement Uncertainty in Experiments

To quantify the reproducibility of the photoluminescence (PL)–based concentration analysis used in the capillary experiments (Fig. 3), we monitored SiV-DND/H_2O and GeV-DND/H_2O signals at two fixed observation points while no optical beams were applied.

Across a continuous 7-hour measurement period, the normalized PL ratios at Position #1 exhibited only modest variations.

For both SiV- and GeV-containing dispersions, the fluctuation amplitude stayed within a band of approximately ±5%. An example time trace is presented in Fig. S6.

Based on these data, we estimate the characteristic experimental uncertainty for the PL-derived concentration ratios in the capillary geometry to be ~5%. This value is used when interpreting the depletion/enrichment behavior in the main text.

Figure S6

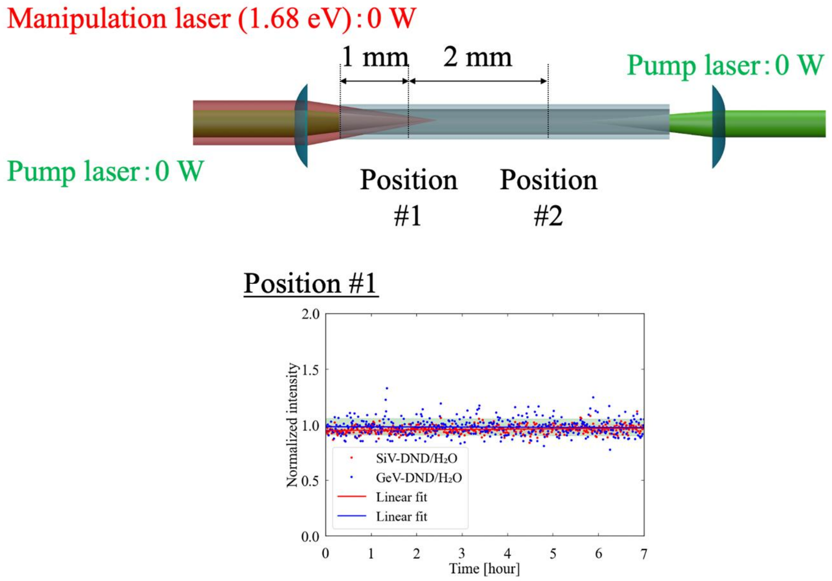

Long-term stability of the PL-derived concentration ratios in the capillary geometry. Time traces of the normalized SiV-DND/H_2O and GeV-DND/H_2O signals at Position #1 are recorded over a continuous 7-hour period with all optical beams blocked. Both signals fluctuate only within approximately ±5% around their mean values, as indicated by the shaded band, establishing the characteristic experimental uncertainty used to interpret depletion and enrichment in the main text.

S7. Estimation of Temperature Inside the Capillary

Local heating within the capillary could potentially induce convection or thermophoretic forces. To evaluate this possibility, we used the temperature dependence of the SiV zero-phonon line (ZPL) as an optical thermometer. The ZPL position in NDs red-shifts with temperature at approximately 0.015 nm/K in the 295–315 K range[10]. We tracked the ZPL peak during irradiation at the two observation points (see Fig. S7). Across the full irradiation interval, the largest measured ZPL displacement was < 0.01 nm, corresponding to an energy shift below ~1 meV.

Using the established calibration, this yields a temperature rise:

$$\Delta T \lesssim 0.7 \text{ K}$$

Such a small temperature increment indicates that:

- significant thermal gradients,
- strong buoyancy-driven flows,
- or thermophoretic drift

are highly unlikely to be the dominant cause of redistribution observed in the capillary. While minor residual flows cannot be ruled out completely, the very small inferred ΔT supports the interpretation that SRF-assisted optical forces, rather than thermal convection, drive the long-range SiV-selective transport.

Figure S7

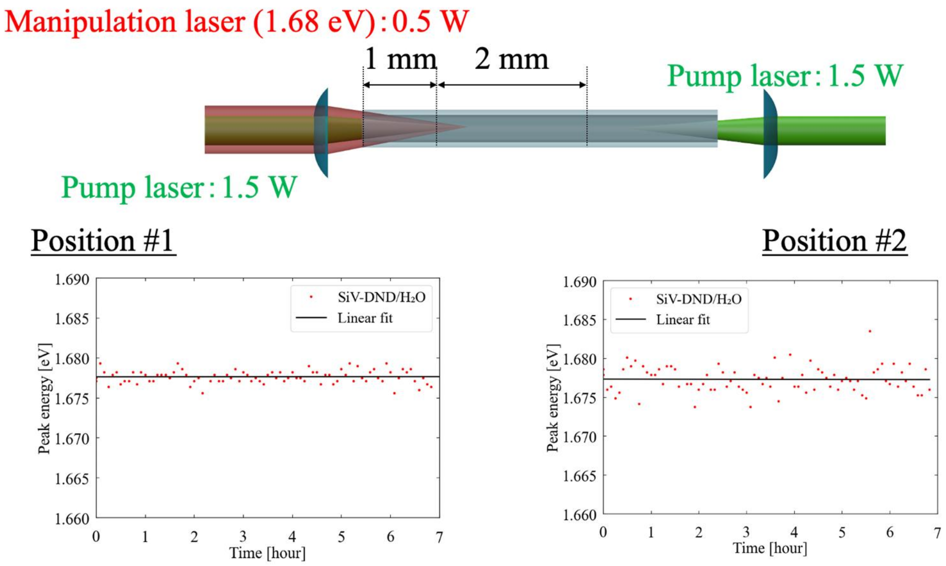

Estimation of the temperature rise inside the capillary from the SiV-ZPL shift. (a) Time evolution of the SiV-ZPL wavelength in the capillary under typical irradiation conditions used in the main text. The observed shift is smaller than 0.01 nm, corresponding to an energy change below ~1 meV and a temperature increase of only a few kelvin, ruling out large thermal gradients or strong thermally driven flows as the main origin of the observed redistribution.

S8. Spectral characteristics and concentration calibration in the glass cell

In the glass-cell configuration, we first confirmed that the spectral signatures of the SiV-DND and GeV-DND dispersions are consistent with those observed in the capillary geometry. Figure S8(a) shows representative PL spectra of SiV-DNDs and GeV-DNDs dispersed in water and measured under the glass-cell excitation conditions employed in the main text. The spectra exhibit the characteristic ZPLs of the SiV^- and GeV^- centers, together with their respective phonon sidebands, demonstrating that the glass-cell excitation and collection scheme preserves the spectral selectivity of the two nanodiamond species.

To enable a quantitative comparison of local concentrations in the glass cell, we next calibrated the PL intensity as a function of nanodiamond concentration. For this purpose, we prepared a series of SiV-DND/H_2O and GeV-DND/H_2O dispersions with known mass concentrations and recorded their PL spectra under identical excitation and detection conditions. The integrated ZPL intensities were normalized by the acquisition time and excitation power to obtain concentration-dependent signals that can be directly compared between different samples.

Figure S8(b) plots the normalized SiV-DND/H_2O signal as a function of the SiV-DND concentration and the corresponding data for GeV-DND/H_2O. In both cases, the data points follow an

approximately linear trend over the concentration range relevant to the main-text experiments, and simple linear fits provide an adequate description of the dependence. This confirms that, under the glass-cell excitation conditions, the normalized SiV-DND/H_2O and GeV-DND/H_2O signals scale linearly with concentration, as stated in the main text. The same calibration procedure is used to convert PL intensities into relative local concentrations in the glass-cell measurements discussed in the main article.

Figure S8

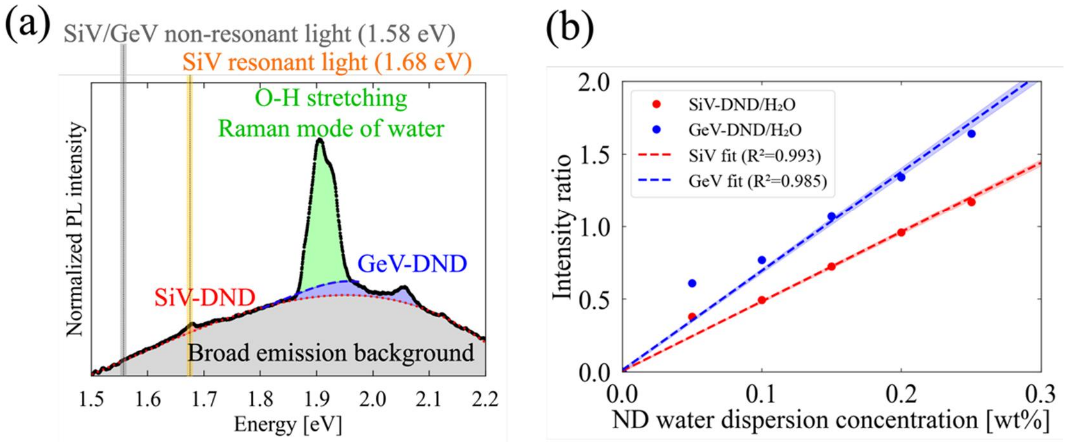

(a) Representative PL spectra of SiV-DND/H_2O and GeV-DND/H_2O dispersions measured in the glass-cell configuration under the excitation conditions used in the main text. The spectra exhibit the characteristic zero-phonon lines (ZPLs) of the SiV^- and GeV^- centers together with their phonon sidebands, confirming that the glass-cell setup preserves the spectral selectivity of the two nanodiamond species. (b) Normalized PL signal as a function of ND concentration for SiV-DND/H_2O and GeV-DND/H_2O dispersions, recorded under identical excitation and detection conditions. Both data sets follow an approximately linear trend over the concentration range relevant to the main-text experiments, demonstrating that the normalized SiV-DND/H_2O and GeV-DND/H_2O signals scale linearly with concentration and can be used as reliable proxies for the local nanodiamond concentrations in the glass cell.

S9. Reproducibility of Capillary and Glass-Cell Experiments

S9.1 Capillary experiments

To quantify the run-to-run reproducibility of the capillary measurements, we repeated the experiment on a series of independently prepared capillaries under nominally identical conditions. In total, we performed N_{cap} =8 runs. For each run, we evaluated the enrichment factor

$$E = \frac{(I_{\mathrm{SiV}}/I_{\mathrm{GeV}})_{end}}{(I_{\mathrm{SiV}}/I_{\mathrm{GeV}})_{initial}}$$

where the SiV and GeV PL intensities were obtained from spatially homogeneous mixtures measured under the same detection conditions as in the main text.

As described in Section S6, repeated PL measurements on a homogeneous dispersion yield an experimental uncertainty of approximately ±0.05 ($1\sigma_E$) in the ratio $I_{\mathrm{SiV}}/I_{\mathrm{GeV}}$. Based on this calibration, we defined a capillary run as "successful" when the final enrichment factor exceeded the +1σ uncertainty band, i.e., when

$$E > E_{th} = 1 + \sigma_E = 1.05$$

Under this criterion, M_{cap}= 5 out of N_{cap} = 8 runs were categorized as successful. In the remaining three runs, the enrichment factors stayed at E = 1.00 within the $\pm\sigma_E$ band, indicating no statistically significant redistribution.

Figure S9(a) summarizes the distribution of E over all capillary runs. The gray histogram shows the full set of N_{cap} measurements, while the red bars highlight the subset of runs that satisfy $E > E_{th}$. The successful runs yield a median enrichment factor of approximately $E_{med} \approx 1.40$, with an interquartile range extending from about 1.22 to 1.79, as indicated by the box-plot overlay. The unsuccessful runs cluster around E ≈ 1.00 within the $\pm\sigma_E$ uncertainty band. This analysis confirms that the subset of capillaries we classify as "successful" exhibits a reproducible enhancement of the SiV/GeV ratio beyond the experimental noise level.

S9.2 Glass-cell experiments

An analogous analysis was carried out for the glass-cell configuration in the standard (non-enhanced) scheme. Here, each run consists of a time series of PL spectra recorded at the irradiation spot of the mixed SiV-DND/GeV-DND dispersion. For each run, we extracted the enrichment factor

$$E = \frac{(I_{\mathrm{SiV}}/I_{\mathrm{GeV}})_{end}}{(I_{\mathrm{SiV}}/I_{\mathrm{GeV}})_{initial}}$$

at the final time point of the measurement.

Figure S9(b) shows the enrichment factor E as a function of the glass-cell run index. Because all runs satisfy E > 1.05, all bars are plotted in orange, and the horizontal dashed line again indicates the threshold. The inset in Fig. S9(b) shows a box plot for all six runs, summarizing the median and spread of E in the standard glass-cell configuration. Compared to the capillary geometry, the glass cell exhibits a narrower distribution of enrichment factors, consistent with the more controlled optical mode structure and alignment in this configuration.

Taken together, the statistics in Fig. S9 demonstrate that the species-selective enrichment observed in both the capillary and glass-cell geometries is reproducible across multiple independent runs. The spread in enrichment factors primarily reflects the sensitivity of the multimode optical field and sample alignment, rather than experimental noise alone.

Figure S9

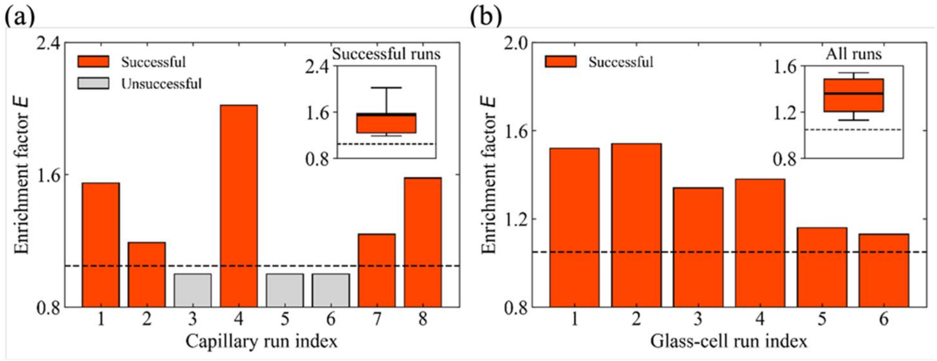

(a) Enrichment factor E plotted as a function of the capillary run index (N_{cap} = 8). Orange bars indicate successful runs with E > 1.05, while light-gray bars indicate unsuccessful runs with $E \leq 1.05$. The horizontal dashed line marks the threshold E_{th} = 1.05. The inset shows a box plot of E for successful runs only, illustrating the median and spread of the enrichment factors that exceed the threshold.

(b) Enrichment factor E plotted as a function of the glass-cell run index in the standard configuration (N_{cell} = 6). All runs satisfy E > 1.05 and are shown as orange bars. The horizontal dashed line again marks E_{th} = 1.05. The inset shows a box plot summarizing the distribution of E over all glass-cell runs.

S10. Control Experiments Excluding Photoionization

A possible alternative explanation for the observed enrichment is photoionization (charge-state conversion) of the defect state under intense optical pumping. Charge conversion is known to affect the electronic configuration and thus the resonance conditions of color centers in diamond (e.g., NV centers). If charge conversion were responsible for the observed increase in PL intensity ratio, an enrichment-like change should appear whenever the illumination conditions that drive ionization are applied, irrespective of whether the stimulated-emission-based manipulation process is enabled.

To test this possibility, we performed three experiments under otherwise identical conditions (same sample, observation volume, acquisition procedure, total illumination time, and power levels; see Materials and Methods). The illumination geometries were:

(a) Pump (counter-propagating) + Manipulation: the standard configuration based on SRF.

(b) Pump only (counter-propagating): identical pump illumination but with the manipulation beam blocked.

(c) Manipulation only: identical manipulation illumination but with the pump beams blocked.

A clear enrichment (increase in the PL intensity ratio/enrichment factor) was observed only in condition (a). In contrast, conditions (b) and (c) showed no measurable enrichment within experimental uncertainty (Fig. S10).

If photoionization were the dominant mechanism, then illumination by either beam alone—particularly the manipulation beam, which is resonant to the SiV centers—should also induce a systematic change in the PL ratio through modification of the defect charge state or local electronic configuration. The absence of any enrichment signal in condition (c), therefore, indicates that charge conversion is not sufficient to produce the observed enrichment. Instead, enrichment appears only when both beams are present simultaneously, which is consistent with a mechanism based on SRF. In other words, the observed selectivity is not explained by an ionization effect driven by high-intensity beams.

These controls strongly suggest that the enrichment is not an artifact of photoionization-driven charge-state conversion but requires the pump + manipulation configuration that establishes the SRF-based manipulation conditions.

Figure S10

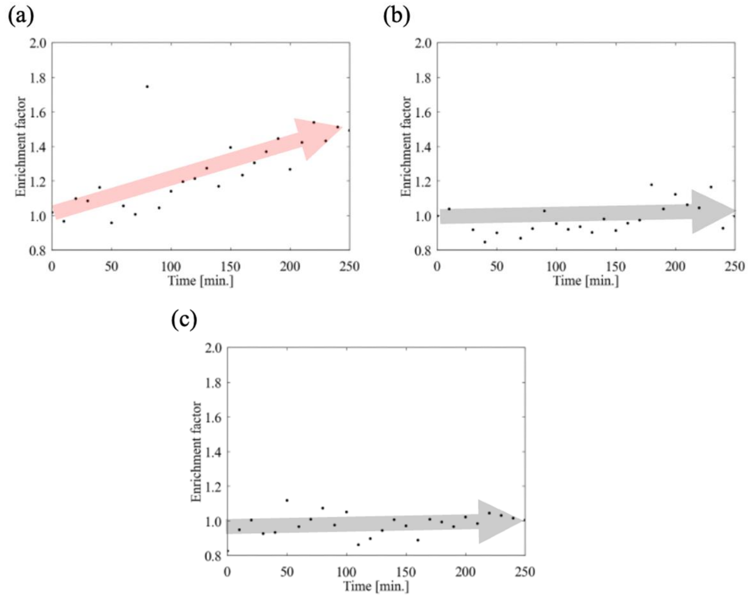

Control experiments addressing photoionization in the glass-cell configuration. Time evolution of the enrichment factor under three illumination conditions: (a) counter-propagating pump beams and a manipulation beam, (b) pump beams only, and (c) manipulation beam only. A clear enrichment is observed only for condition (a), whereas no systematic increase is seen for (b) or (c) within experimental uncertainty.

References

(1) Iida, T.; Ishihara, H. Theory of resonant radiation force exerted on nanostructures by optical excitation of their quantum states: From microscopic to macroscopic descriptions. *Phys. Rev. B* **2008**, *77*, 245319.

(2) Bavli, R.; Heller, D.; Band, Y. Nonlinear optical response of three-level systems. *J. Chem. Phys.* **1989**, *91*, 6714–6727.

(3) So, F. T.-K.; Hariki, N.; Nemoto, M.; Shames, A. I; Liu, M.; Tsurui, A.; Yoshikawa, T.; Makino, Y.; Ohori, M.; Fujiwara, M.; Herbschleb, E. D.; Morioka, N.; Ohki, I.; Shirakawa, M.; Igarashi, R.; Nishikawa, M.; Mizuochi, N. Small multimodal thermometry with detonation-created multi-color centers in detonation nanodiamond. *APL Mater.* **2024**, *12*, 051102.

(4) Becker, J. N.; Becher, C. Coherence Properties and Quantum Control of Silicon Vacancy Color Centers in Diamond. *Phys. Status Solidi A* **2017**, *214*, 1700586.

(5) Ulbricht, R.; Dong, S.; Gali, A.; Meng, S.; Loh, Z.-H. Vibrational relaxation dynamics of the nitrogen-vacancy center in diamond. *Phys. Rev. B* **2018**, *97*, 220302.

(6) Makino, Y.; Saito, Y.; Takehara, H.; Tsurui, A.; Okuyama, N.; Ashida, M. Effect of Particle Size on the Optical Properties of Silicon-Vacancy Centers in Nanodiamonds Fabricated by a Detonation Process. *Phys. Status Solidi A* **2022**, *219,* 2200342.

(7) Barton, J. P.; Alexander, D. R. Fifth - order corrected electromagnetic field components for a fundamental Gaussian beam. *J. Appl. Phys.* **1989**, *66*, 2800-2802.

(8) Makino, Y.; Saito, Y.; Minowa, Y.; Tsurui, A.; Kishino, Y.; Kouuchi, T.; Takeuchi, Y.; Yamagishi, G.; Ashida, M. Optical properties of negatively charged germanium-vacancy centers in detonation nanodiamonds with an average single-digit nanometer particle size. *Jpn. J. Appl. Phys.* **2024**, *63*, 035003.

(9) Nishikawa, M.; Liu, M.; Yoshikawa, T.; Takeuchi, H.; Matsuno, N.; Komatsu, N. Thorough elucidation of synthesis and structure of poly (glycerol) functionalized nanodiamonds. *Carbon* **2023**, *205*, 463-474.

(10) Fujiwara, M.; Uchida, G.; Ohki, I.; Liu, M.; Tsurui, A.; Yoshikawa, T.; Nishikawa, M.; Mizuochi, N. All-optical nanoscale thermometry based on silicon-vacancy centers in detonation nanodiamonds. *Carbon* **2022**, *198*, 57-62.

(11) Yoshikawa, T.; Liu, M.; Chang, S. L. Y.; Kuschnerus, I. C.; Makino, Y.; Tsurui, A.; Mahiko, T.; Nishikawa, M. Steric Interaction of Polyglycerol-Functionalized Detonation Nanodiamonds. *Langmuir* **2022**, *38*, 661-669.

(12) Basiuk, E. V.; Santamaría-Bonfil, A.; Meza-Laguna, V.; Gromovoy, T. Y.; Alvares-Zauco, E.; Contreras-Torres, F. F.; Rizo, J.; Zavala, G.; Basiuk, V. A. Solvent-free covalent functionalization of nanodiamond with amines. *Appl. Surf. Sci.* **2013**, *275*, 324-334.

(13) Bolshakov, A.; Ralchenko, V.; Sedov, V.; Khomich, A.; Vlasov, I.; Khomich, A.; Trofimov, N.; Krivobok, V.; Nikolaev, S.; Khmelnitskii, R.; Saraykin, V. Photoluminescence of SiV centers in single crystal CVD diamond in situ doped with Si from silane. *Phys. Status Solidi A.* **2015**, *212*, 2525-2532.
